# New landscapes and horizons in hepatocellular carcinoma therapy

**DOI:** 10.18632/aging.102777

**Published:** 2020-02-04

**Authors:** Melchiorre Cervello, Maria R. Emma, Giuseppa Augello, Antonella Cusimano, Lydia Giannitrapani, Maurizio Soresi, Shaw M. Akula, Stephen L. Abrams, Linda S. Steelman, Alessandro Gulino, Beatrice Belmonte, Giuseppe Montalto, James A. McCubrey

**Affiliations:** 1 Institute for Biomedical Research and Innovation, National Research Council (CNR), Palermo, Italy; 2 Department of Health Promotion Sciences Maternal and Infantile Care, Internal Medicine and Medical Specialties, University of Palermo, Palermo, Italy; 3 Department of Microbiology and Immunology, Brody School of Medicine at East Carolina University, Greenville, NC 27858, USA; 4 Tumour Immunology Unit, Human Pathology Section, Department of Health Science, University of Palermo, Palermo, Italy

**Keywords:** HCC, targeted therapy, immunotherapy, aging, cancer

## Abstract

Hepatocellular carcinoma (HCC), is the sixth most frequent form of cancer and leads to the fourth highest number of deaths each year. HCC results from a combination of environmental factors and aging as there are driver mutations at oncogenes which occur during aging. Most of HCCs are diagnosed at advanced stage preventing curative therapies. Treatment in advanced stage is a challenging and pressing problem, and novel and well-tolerated therapies are urgently needed. We will discuss further advances beyond sorafenib that target additional signaling pathways and immune checkpoint proteins. The scenario of possible systemic therapies for patients with advanced HCC has changed dramatically in recent years. Personalized genomics and various other omics approaches may identify actionable biochemical targets, which are activated in individual patients, which may enhance therapeutic outcomes. Further studies are needed to identify predictive biomarkers and aberrantly activated signaling pathways capable of guiding the clinician in choosing the most appropriate therapy for the individual patient.

## INTRODUCTION

Liver cancer accounts for more than 840,000 new cases and about 780,000 deaths annually, representing the sixth most common cancer and the fourth most frequent cause of cancer death, respectively, globally [1]. Hepatocellular carcinoma (HCC) is the primary cancer most commonly encountered in the liver (comprising about 90% of cases). The main risk factors for HCC are chronic infection with hepatitis B (HBV) or C (HCV) viruses, alcohol abuse, and non-alcoholic fatty liver disease (NAFLD).

In recent years, the prognosis of patients with HCC at early- or intermediate-stages has significantly improved because of advancements in diagnosis and curative treatments. However, HCC prognosis is still extremely poor, since it is highly resistant to curative treatments, such as surgical resection or ablation, and therefore, 70% of patients have tumor recurrence within 5 years.

Most of HCCs are diagnosed at advanced stage when curative therapies are not feasible. Nevertheless, systemic therapy in the advanced stage was quite limited until 2007, when the scenario changed drastically by the introduction of the molecular-targeted agent sorafenib, an oral multi-kinase inhibitor targeting RAF kinase, as well as vascular endothelial growth factor receptors (VEGFRs) and additional kinases.

However, currently systemic sorafenib monotherapy has modest clinical benefits, and it has relatively severe side effects. Thus, HCC treatment in advanced stage is a challenging and pressing problem, and novel and well-tolerated therapies are urgently needed for this disease.

HCC development is a multifactorial and a complex multistep process. In fact, a series of genomic and epigenomic alterations, resulting in progression from pre-cancerous lesions, which develop in cirrhotic liver, to the so-called dysplastic nodules, to HCC has been documented. HCC is a disease of aging and most victims are adults and not children [2].

The increase in age is a very well known risk factor for the development of HCC, but the increase in its incidence in the elderly cannot be only related to the aging of the general population. The reasons for this progressive aging of the population with HCC are mainly related to the epidemiological variations of its main risk factors [3].

It is known that HCC occurs predominantly (approximately 90%) on liver cirrhosis, and both these diseases share the same risk factors. In particular, HBV and HCV viruses, alcohol abuse, and in some geographical areas aflatoxin exposure are considered the most frequent. NAFLD, autoimmune and cholestatic diseases, while predisposing to its onset, appear to have a minor epidemiological role. In this regard, however, it should be noted that non-alcoholic steatohepatitis (NASH), most likely in the coming years, will be the main risk factor [4–7].

The increase in age at diagnosis of HCC depends largely on the population and risk factors; patients with HCC living in geographic areas with high incidence rates have a younger age than those living in areas with lower incidence [3, 8]. This behaviour depends very much on the age at which the viral infection is contracted and on the duration of the infection, in endemic areas patients come in contact with viruses at birth, or while infant, therefore the appearance of liver cirrhosis and consequently of HCC occurs at a younger age [7, 9, 10].

Several studies comparing the age at diagnosis of HCC over the last decade compared to that of the previous decades have reported, especially in HCV-infected subjects, a significant aging of patients passing from an average age at diagnosis of about 60-65 years in the last decade of the last century to 70-72 in the last ten years [11–13]. In Italy, the ARTIUM report demonstrated that the frequency of HCC increases in relation to the average age of the population [14].

It should also be emphasized how the health prevention campaigns and antiviral therapies have affected these changes. In fact, the spread of HBV vaccination and the careful screening of HCV, the use of disposable needles and syringes, and changes in various medical procedures, have helped to reduce new viral infections [7]. Today, the subjects with chronic liver diseases are mostly those who contracted the disease in the 1960’s and 1970’s. [15]. Furthermore, antiviral therapies against HBV and HCV have contributed to the aging of patients with HCC contributing to cause it to arise later rather than to abolish its risk at all [16].

In contrast to the reduction of the role of HBV and above all of HCV, which is taking place in some countries, the role of HCC related to NAFLD is becoming increasingly evident. Even in these cases, it seems that HCC is diagnosed at an age greater than 65-70 years, sometimes not on cirrhotic liver and with very severe prognosis [7, 17].

## MOLECULAR ALTERATIONS IN HCC

Several alterations have been detected in HCC, including CTNNB1 (β-catenin), AT-Rich Interaction Domain 1A (ARID1A), ARID1B, AXIN, telomerase reverse transcriptase (TERT), c-MYC, epidermal growth factor (EGF), hepatocyte growth factor (HGF), as well as RAS and TP53 mutations, fibroblast growth factor 19 (FGF19) amplification, cyclin dependent kinase inhibitor 2A (CDK2A) downregulation and insulin growth factor 2 (IGF2) overexpression due to epigenetic modifications [18]. These molecular alterations ultimately lead to activation of signaling pathways, which have pivotal roles in HCC tumorigenesis.

Unfortunately, there does not appear to be a unique signaling pathway which is predominantly altered in HCC [18], this likely results from the inter-tumor molecular heterogeneity observed in HCC [19, 20]. There are several molecular HCC subtypes, presenting different molecular aberrations, responsible for cell proliferation and survival, while other alterations, which are present in almost all HCCs, involve limitless replicative potential, angiogenesis, resistance to anti-proliferative signals and checkpoint controls [18, 21]. Only a minority of alterations found in HCC are targetable with drugs currently available, most of them are not clinically-actionable today.

However, various approaches have been used or are being developed for HCC treatment. Therapies currently used to treat HCC are fundamentally based on pharmacological approaches, which include: traditional cytotoxic chemotherapeutic drugs, small-molecule inhibitors, such as sorafenib, and monoclonal antibodies (MoAbs), which target a specific molecule(s) implicated in HCC pathogenesis. In addition, combination therapies of small-molecule inhibitors with traditional cytotoxic drugs, or with another inhibitor that acts on a specific molecule in the same or in different signal transduction pathways, or with MoAbs, are also being tested.

Until now, the US FDA has approved over 20 small-molecule inhibitors and more than 65 MoAbs for clinical treatment of cancer. Some examples of small-molecule inhibitors and MoAbs, which we discuss in this review, include agents acting on the following signaling pathways: RAS/RAF/mitogen-extracellular activated protein kinase kinase (MEK)/extracellular signal-regulated kinase (ERK); phosphatidylinositol-3-kinase (PI3K)/AKT/mammalian target of rapamycin (mTOR); EGF/EGF receptor (EGFR); platelet-derived growth factor (PDGF)/PDGF receptor (PDGFR); vascular endothelial growth factor (VEGF)/VEGF receptor (VEGFR); FGF19/FGF receptor 4 (FGFR4); transforming growth factor β receptor (TGFβR); c-MET; cyclin-dependent kinases (CDKs); aurora kinases (AURKs); histone deacetylases (HDACs); programmed death 1 (PD-1); cytotoxic T lymphocyte antigen-4 (CTLA-4).

Recently, the development of immune-oncologic agents has opened new therapeutic opportunities for cancer treatment, including HCC. These agents target mainly two immune checkpoints, PD-1 and CTLA-4, which are negative regulators of T-cell immune function, resulting in reversal of immune exhaustion, and activation of immune response. Immune checkpoint inhibitors (ICIs) have been shown to be effective for the treatment of various cancer types including: non-small cell lung cancer (NSCLC), melanoma, renal cell carcinoma and advanced urothelial bladder cancer. There is high expectation that these new drugs may help to develop new therapies for HCC, used as single agent, or in combination with each other or with molecular targeted agents.

**Figure 1:**
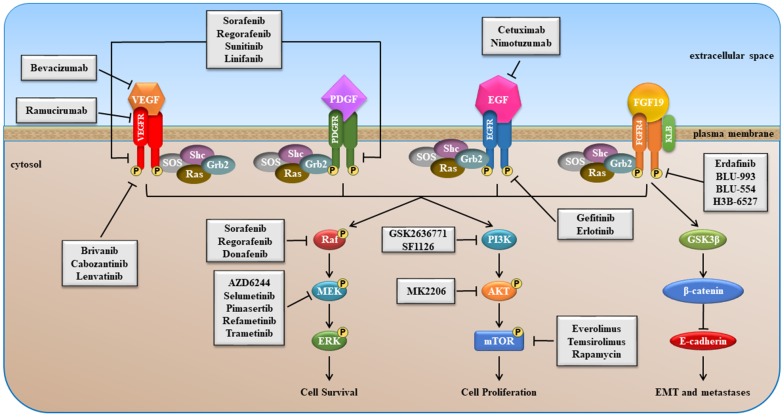
**Schematic overview of VEGFR, PDGFR, EGFR and FGFR signaling pathways stimulated after binding of growth factor (GF).**

**Figure 2:**
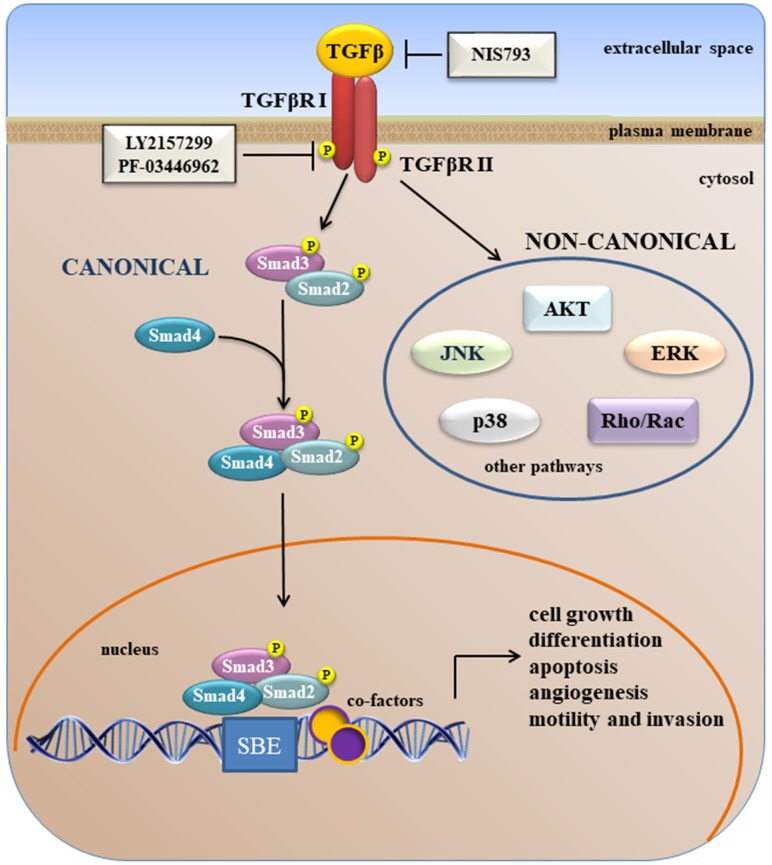
**A simplified overview of canonical and non-canonical TGFβ signaling.**

**Figure 3:**
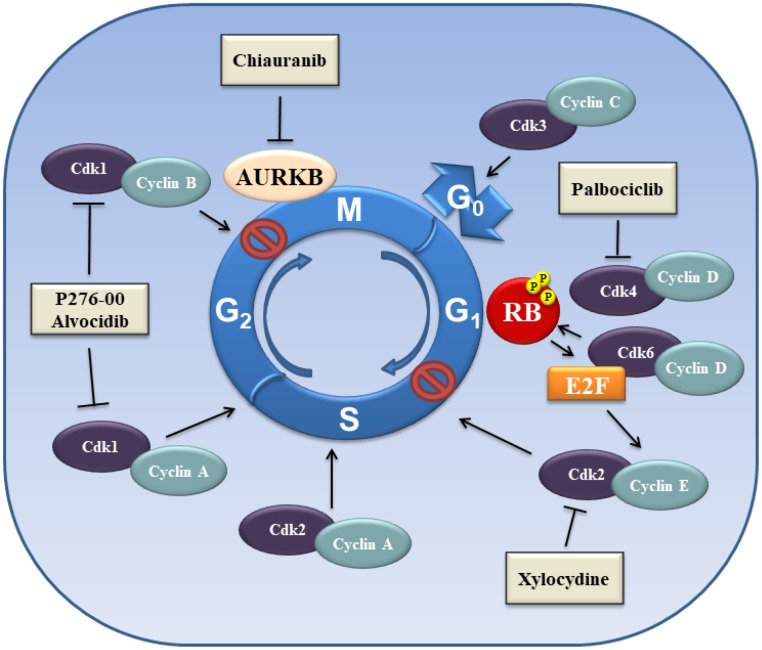
**Schematic overview of proteins involved in the control of cell cycle.**

**Figure 4:**
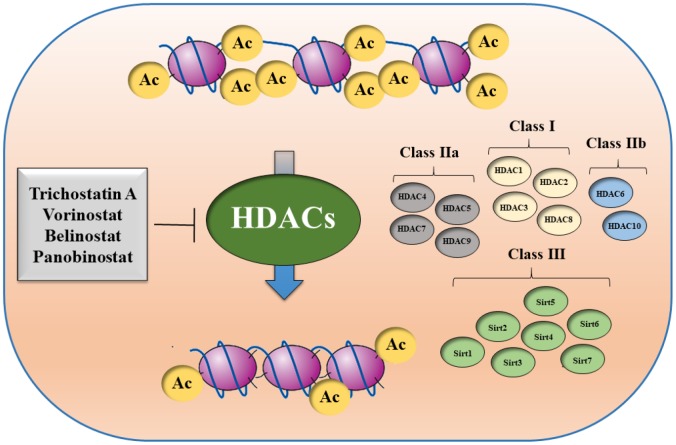
**Schematic overview of different HDAC classes.**

**Figure 5:**
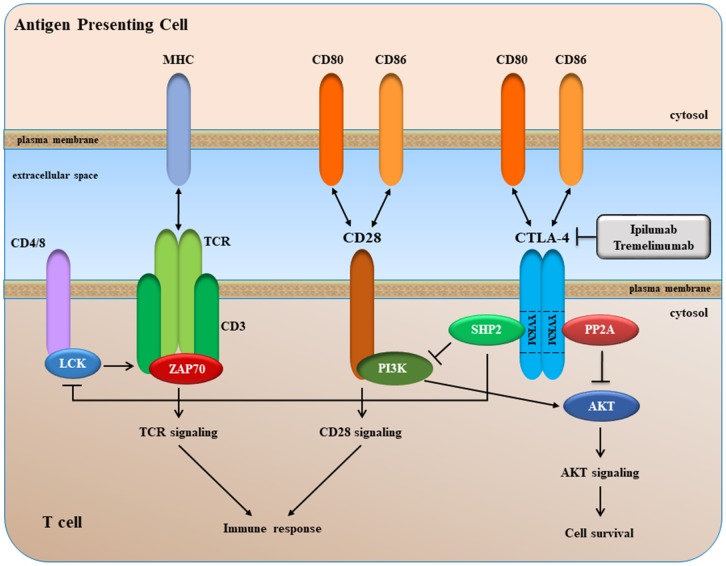
**A simplified overview of CTLA-4 signaling.**

**Figure 6:**
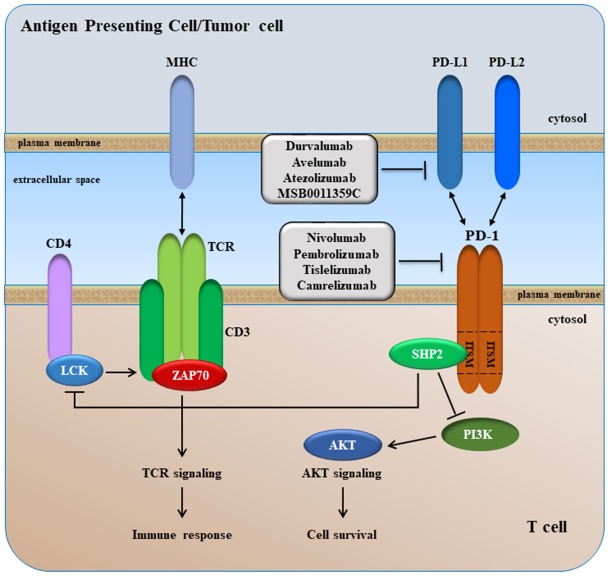
**A simplified overview of PD-1 signaling.**

In this review, we summarize the major signaling pathways and molecular targets involved in HCC pathogenesis (Figures 1–6) and discuss the current status and the prospects for the near future of systemic therapies, including immunotherapies, for HCC management (Tables 1 and 2). We performed Medline searches from 2000-2019 and ClinicalTrials.gov to obtain information for this review.

## SIGNALING PATHWAYS IN HCC

Several components of signaling pathways, such as RAS/RAF/MEK/ERK, PI3K/AKT/mTOR, VEGF/ VEGFR, EGF/EGFR, etc. are promising targets in HCC [18, 22, 23]. Schematic overviews of the most important signaling pathways, as well as sites of intervention with small molecule inhibitors and MoAbs, are presented in Figures 1–6. Most small-molecule inhibitors act on a single target (e.g., erlotinib, BLU-554, everolimus and others), while some are promiscuous (e.g., sorafenib, regorafenib, lenvatinib and others), i.e. they act simultaneously on more than one molecule, however, this multiple targeting could increase their therapeutic efficacy (Figure 1).

Activation of these pathways play key roles in the control of cell proliferation and in the resistance to apoptosis of tumor cells. Moreover, they contribute to stimulation of tumor angiogenesis, promotion of cellular invasiveness and metastasis.

In the last 20 years, many advances have been made in understanding the interactions among pathway components, as well as the mechanisms by which different pathways interact with each other. Furthermore, numerous discoveries have been made into the mechanisms by which mutations of some components of these pathways are able to cause aberrant signaling, loss of control of cell proliferation and ultimately alterations in response to targeted therapies.

## Background and strategies based on targeting EGF/EGFR

EGF/EGFR signaling (Figure 1) is dysregulated in many cancers including: breast, gastric, HCC, lung and ovarian [24]. The EGFR signaling pathway has been a key therapeutic target for decades now. Various approaches have been developed to target the EGF/EGFR pathway including small molecule tyrosine kinase domain inhibitors as well as MoAbs [25]. The effects of targeting the EGFR in HCC with small molecule kinases inhibitors, such as erlotinib and gefitinib, have been evaluated [26, 27]. The EGFR inhibitor suppressed liver fibrosis and the development of HCC [26].

In addition, the effects of anti-EGFR MoAbs, such as cetuximab (Erbitux®, Bristol-Myers Squibb; Merck Serono) and nimotuzumab, have been examined on HCC. In one study, an elderly HCC patient treated with nimotuzumab resulted in a complete remission [28].

Clinical trials with EGFR inhibitors and HCC and other cancers have been performed and some are in progress [18]. Gefitinib, by itself was not observed to be effective in treatment of HCC [29]. The effects of combining an EGFR inhibitor and an inhibitor of another pathway, or by a chemotherapeutic drug, or an immunotherapeutic approach are being examined. They are listed in Table 1. The effects of combining the EGFR inhibitor erlotinib with the chemotherapeutic drug docetaxel have been examined in the NCT00532441 phase II clinical trial [30]. In this study, combining erlotinib with docetaxel did not appear to result in enhancement of survival in comparison to patients treated with erlotinib by itself. The effects of combining the VEGFA inhibitor bevacizumab and erlotinib have been examined in the NCT0336591 phase II clinical trial [31]. Unfortunately, this study observed that combining erlotinib with bevacizumab only resulted in a minimal activity in patients with advanced HCC. The authors have suggested that the inclusion of molecularly-selected HCC patients with particular mutation profiles could have enhanced the outcome.

**Table 1:** Different molecular targeted agents, and their combinations with other agents under clinical evaluation in HCC (as of December 2019)^1^.

**Target**	**Agent(s) (trade name; code name; Company)**	**Phase**	**Status**	**Clinicaltrials.gov Identifier**
*EGFR Inhibitors*				
**EGFR/HER2**	FATE-NK100 + either Cetuximab (Erbitux®; Bristol-Myers Squibb, Merck Serono) or Trastuzumab (Herceptin®; Roche)	I	recruiting	NCT03319459
*MEK1 Inhibitors*				
**MEK1**	Trametinib (Mekinist®; GlaxoSmithKline)	I	recruiting	NCT02070549
**MEK1, RAF and other kinases**	Sorafenib (Nexavar®, Bayer) + Trametinib	I	active, not recruiting	NCT02292173
*PI3K Inhibitors*				
**PI3K, PD1**	SF1126 and Nivolumab	I	active, not recruiting	NCT03059147
**PI3K-β and other kinases**	GSK2636771 and other kinase inhibitors	I	recruiting	NCT02465060
*mTORC1*				
**mTORC1**	Sirolimus and liver transplantation	II/III	not recruiting yet	NCT03500848
**mTORC1 and RAF kinases**	Temsirolimus and sorafenib	II	active, not recruiting	NCT01687673
**mTORC1 and estrogen receptor**	Everolimus and leuprolide and letrozole	II	active, not recruiting	NCT01642186
*Dual mTORC1/mTORC2*				
**mTORC1, mTORC2**	CC-223	I	recruiting	NCT03591965
*VEGFR/PDGFR inhibitor*				
**VEGFR**	Cabozantinib (XL184; Cabometyx®, Cometriq®, Exelixis Inc.)	III	active, not recruiting	NCT01908426
	Lenvatinib (Lenvima®; Eisai),	III	active, not recruiting	NCT01761266
	Ramucirumab (LY3009806, IMC-1121B, Cyramza®; Eli Lilly and Company)	III	recruiting	NCT02435433
**VEGFR, PDGFR**	Regorafenib (Stivarga®, Bayer)	III	completed	NCT01774344
*FGFR4*				
**FGFR4**	BLU-554 (Blueprint Medicines Corporation)	I	recruiting	NCT02508467
	H3B-6527 (H3 Biomedicine Inc.)	I	recruiting	NCT02834780
*TGFβs*				
**TGF β**	NIS793 (Novartis Pharmaceuticals) + PDR001 (Novartis Pharmaceuticals)	I/Ib	recruiting	NCT02947165
**TGFβRI**	LY2157299 (Galunisertib®; Eli Lilly) + Sorafenib	II	active, not recruiting	NCT02178358
	LY2157299 + Nivolumab (Opdivo®; Bristol-Myers Squibb Pharma EEIG)	II	active, not recruiting	NCT02423343
	LY2157299 + Radiation: Stereotactic Body Radiotherapy (SBRT)	II	active, not recruiting	NCT02906397
*CDKs inhibitor*				
**CDK4, CDK6**	Palbociclib (Ibrance®; Pfizer)	I/II	active, not recruiting	NCT01356628
*Combination of HCDCi with sorafenib*				
**Class I, II and IV**	Vorinostat (‎Zolinza ®; Merck Sharp Dohme) + Sorafenib	I	completed, no results posted	NCT01075113
*Multi-target inhibitor*				
**AURKB, VEGFR2, VEGFR1, VEGFR3, PDGFRα, c-KIT, CSF-1R**	Chiauranib (Shenzhen Chipscreen Biosciences, Ltd., China)	I	recruiting	NCT03245190

^1^More through discussion of the results of clinical trials, when available, is presented in the text of this manuscript.

An ongoing HCC clinical trial (NCT03329459) consists of determining the effects of combining FATE-NK100, an allogeneic donor-derived natural killer (NK) cell based cancer immunotherapy, with the anti-EGFR MoAb cetuximab. FATE-NK100 consists of adaptive active memory NK expressing the maturation marker CD57. No results from this study appear to have been published yet (January 2020).

## Background and strategies based on targeting PDGF/PDGFR and VEGF/VEGFR

Angiogenesis and metastases are two of the major obstacles for overcoming the challenges of cancer treatment. The molecular basis of these processes revealed alterations in different molecular signaling pathways, including the PDGF and VEGF pathways.

PDGFR is a member of the class III family of receptors with tyrosine kinase activity (RTK) [32]. Two different monomeric forms of PDGFR are known, namely PDGFRα and PDGFRβ. Structurally, they are characterized by an extracellular domain containing five immunoglobulin-like motifs, a single trans-membrane region, and an intracellular domain with tyrosine-kinase (TK) activity. The binding of PDGF to its receptor induces PDGFR homo- or heterodimerization that results in auto-phosphorylation of specific tyrosine residues present in its intracellular domain. This results in conformational changes of the intracellular domain of receptor that is necessary for its complete activation, and to induce binding and phosphorylation of Src homology-2 (SH2)-domain containing–molecules, leading to activation of various signaling pathways, such as RAS/RAF/MAPK and PI3K/PDK1/AKT signaling (Figure 1). Alterations of PDGF/PDGFR pathway are reported in several malignancies and expression of PDGFR is associated with poor prognosis and metastatic potential in different tumor types, such as breast and gastric cancer [33, 34].

The VEGFR family consists of three monomeric forms known as VEGFR-A, -B and -C. The family members are characterized by an extracellular region containing seven immunoglobulin-like domains, a single trans- membrane region and an intracellular domain with TK activity. Activation of VEGFR signaling requires the binding of its specific ligand (VEGFs) to the extracellular domains and the ligand-induced dimerization or multimerization of receptor monomers. Once activated, the receptor interacts with SH2 domain of its molecular targets and induces phosphorylation and activation of different molecules, such as ERK and AKT, switching on VEGFR signaling inside the cells (Figure 1). Aberrant expression of VEGF and VEGFR are observed in several cancer types, such as gastric, pancreatic, breast and colorectal cancer, where they mediate tumor angiogenesis and expansion [35–38].

VEGFR and PDGFR represent promising targets for treatment of several malignancies. Many authors have reported that overexpression of PDGFRs and VEGFRs, and their ligands PDGFs and VEGFs, frequently occurs in HCC and is associated with poor prognosis and worse overall survival (OS) [39–43]. In addition, overexpression of these factors, and their specific receptors, is linked with recurrence of liver cancer after tumor surgical resection [39, 42]. Several clinical studies have been conducted and are ongoing to evaluate the effects of inhibition of VEGF/VEGFR and PDGF/ PDGFR pathways in management of HCC treatment.

Sorafenib (BAY 43-9006; Nexavar®, Bayer), an oral multi-kinases inhibitor targeting VEGFR, PDGFR and other kinases, represents first-line systemic therapy available for patients with advanced HCC and not eligible for tumor resection or liver transplantation. The international phase III study known as Sorafenib Hepatocellular Carcinoma Assessment Randomized Protocol (SHARP) in patients with advanced HCC, who had not received any prior drug, demonstrated that, in comparison to placebo, sorafenib administration resulted in increased OS (10.7 months vs 7.9 months) and median time to progression (TTP) (5.5 versus 2.8 months) (NCT00105443) [44]. Unfortunately, it was demonstrated that sorafenib administration was not able to prolong patient’s OS over one year and caused several (even if manageable) side effects, such as hand-foot skin reaction, hypophosphatemia and weight loss [44]. Thus, new therapeutic approaches are needed for improvement of HCC treatment.

Regorafenib (Stivarga®, Bayer), structurally similar to sorafenib, is an oral multi-kinases inhibitor with pharmacological activity against factors involved in tumor angiogenesis (such as VEGFRs), in tumor cell proliferation (such as c-KIT, RAF and RAS) and tumor microenvironment (such as PDGFRs and FGFRs). The international phase III RESORCE study (NCT01774344) (Table 1), including patients with advanced HCC that received sorafenib as first-line treatment, demonstrated that, in comparison to the control group, patients who received regorafenib had benefits in terms of OS (10.6 months vs 7.8 months; HR 0.63; 95% CI: 0.50–0.79; *p*<0.0001) and median TTP (3.6 months vs 1.5 months). In subsequent analyses of clinical outcomes obtained from RESORCE study, Finn et al. [45] revealed that the median time of OS from start of sorafenib treatment to death, was notably prolonged in regorafenib group compared to control group (26.0 months vs 19.2 months). However, correlation of serum levels of the prognostic markers alpha-fetoprotein (AFP) and c-MET with clinical outcomes obtained from RESORCE study revealed that regorafenib benefits were independent from AFP and c-MET protein levels for prediction of both OS and median TTP [46].

Sunitinib (Sutent®, Pfizer Inc.) is another multi-kinases inhibitor with antitumor and anti-angiogenic activities that acts against a wide range of RTK partially overlapping with sorafenib targets, such as VEGFR and PDGFR. Several preclinical studies demonstrated that sunitinib was able to delay cell growth and proliferation of endothelial cells, inhibiting new vessels formation and causing tumor regression in *in vivo* models of murine xenografts derived from different tumor cell lines, such as gastric and colon-rectal cancer [47]. Different phase II clinical trials displayed antitumor effects of sunitinib monotherapy in patients with advanced HCC with manageable adverse effects [48–50]. Thus, a phase III study (NCT00699374) was performed to compare effects of sunitinib and sorafenib administration in patients with unresectable HCC. However, this trial was rapidly interrupted because of the lack of purposed sunitinib administration advantages in OS of HCC patients. OS from sunitinib administration were not superior, or equivalent, but significantly inferior to sorafenib treatment in HCC patients enrolled in this study.

Similarly, linifanib (ABT-869), a selective inhibitor of all VEGFRs and PDGFRs, showed promising antitumor effects in phase II clinical trials [51], but failed in phase III study (NCT01009593) the first endpoint when compared with sorafenib treatment, and revealed equal advantages in terms of OS (9.1 months vs 9.8 months). Moreover, most of patients that received linifanib had more serious side effects than those observed in the group of patients receiving sorafenib [52].

Brivanib (BMS-582664; Bristol-Myers Squibb) is a TK inhibitor (TKI) which targets VEGFRs and FGFRs pathways. Several phase II clinical trials in patients with advanced HCC revealed promising antitumor activities of the drug, used as both first-line treatment or in second-line treatment, in patients which received prior sorafenib administration [53, 54]. However, different randomized open-label phase III studies (BRISK) did not yield satisfactory results of brivanib treatment in HCC patients. In particular, in the BRISK-FL study (NCT00858871), brivanib failed to achieve the first endpoint of non-inferior OS when compared to sorafenib (9.1 months vs 9.5 months), both drugs had similar antitumor activity with comparable safety profile [55]. In the BRISK-PS study (NCT00825955), brivanib was orally administered in patients that previously received sorafenib treatment. In this cohort of patients, brivanib treatment did not yield any advantages in terms of OS and caused treatment-related side effects in 23% of patients [56]. Similar negative results and no effects in improvement of OS were obtained from another international randomized phase III study (NCT00908752) in which brivanib was used as adjuvant in transarterial chemoembolization (TACE) treatment of patients with intermediate stage of unresectable HCC [57].

Cabozantinib (XL184; Cabometyx®, Cometriq®, Exelixis Inc.) is a small oral TK inhibitor which may inhibit several TKs frequently overexpressed in several malignancies, such as MET, RET and VEGFRs. Dual blockade of MET and VEGFR2 mediated by cabozantinib treatment significantly reduced HCC cell proliferation and metastatic potential both *in vitro* and *in vivo* xenograft models [58]. A phase II open-label discontinued clinical study was conducted with nine types of solid tumor patients, including HCC. This study (NCT00940225) observed that, even if no significant differences were revealed between placebo and cabozantinib treatment groups in terms of progression free survival (PFS), cabozantinib reduced tumor progression, induced disease stabilization, with an associated reduction of serum levels of AFP in more than 50% of patients. A phase III randomized double-blind study (CELESTIAL; NCT01908426), conducted with 707 patients with advanced HCC pre-treated with sorafenib, revealed encouraging results regarding the clinical activity of the drug. Cabozantinib treatment resulted in longer OS (10.2 months vs 8 months) and PFS (5.2 months vs 1.9 months) than placebo. However, cabozantinib did induce two-times more grade 3 and 4 adverse events than placebo (68% vs 36%) [59]. Nevertheless, on 14 January 2019, the US FDA approved cabozantinib for patients with HCC who have been previously treated with sorafenib. The recommended dose is 60 mg once a day.

Another orally available small TK inhibitor, lenvatinib (Lenvima®; Eisai), was evaluated in a phase III study (REFLECT; NCT01761266) as first-line treatment in patients with advanced HCC, and showed non-inferior clinical activity compared to sorafenib in terms of median OS (13.6 months vs 12.3 months), and a statistically significant improvement in PFS, and comparable toxicity profile [60]. Based on REFLECT study, lenvatinib has been approved by US FDA on August 2019, for first-line treatment of patients with unresectable HCC.

In addition to small TKIs, there is another class of molecules with anti-angiogenic activity that includes MoAbs against VEGF or VEGFR, such as bevacizumab (Avastin®; Genentech/Roche) and ramucirumab (LY3009806, IMC-1121B, Cyramza®; Eli Lilly and Company), respectively.

In particular, bevacizumab, is a MoAb against VEGF, that is able to block interaction between VEGF and its receptor VEGFR, reducing VEGFR activation and inhibiting angiogenesis. Despite encouraging results obtained both *in vitro* and *in vivo* in xenograft models derived from HCC cell lines [61, 62], several phase II clinical studies using bevacizumab alone, or combined with other drugs, or as adjuvant in TACE-treated patients, failed to demonstrate improvement [63–67].

Ramucirumab is a humanized MoAb against extracellular domain of VEGFR-2. A phase II clinical trial (NCT00627042) involving patients with advanced HCC revealed that intravenous administration of ramucirumab in monotherapy yielded notable results in terms of median OS (12 months) and median PFS (4.0 months). These positive results prompted initiation of the phase III REACH clinical trial [68] in which ramucirumab and placebo were used as second-line treatment in patients that previously received sorafenib. Despite manageable side effects, ramucirumab did not appear to improve significantly median OS compared to placebo (9.2 months vs 7.6 months) and PFS (2.9 vs 2.1). However, efficacy of ramucirumab was observed in a subgroup of patients with elevated levels of AFP (at least 400 ng/ml). In the REACH-2 phase III clinical trial (NCT02435433), ramucirumab was administered as a second-line treatment after sorafenib in patients with advanced HCC and had at least 400 ng/ml of serum AFP. The study reached the primary endpoint with improvement of median OS (8.5 vs 7.3) and PFS (2.8 vs 1.6) and represents the first phase III study with encouraging results concerning the effectiveness of ramucirumab in second-line therapy of HCC [69]. Based on these results, on May 10, 2019, the US FDA approved ramucirumab for treatment of HCC patients for patients who have been previously treated with sorafenib and have an AFP of ≥ 400 ng/mL.

## Background and strategies based on targeting FGF19/FGFR4

FGF19 is a component of the large family of FGFs that are involved in regulating many biological processes, including cell growth and survival, metabolic and neuronal signaling. FGF19 acts as a hormone with endocrine functions. Its actions are mediated by its binding to specific receptor FGFR4, highly expressed in the liver. Although, FGF19 can bind independently to its receptor FGFR4, the presence of its co-activator beta-Klotho (KLB), a trans-membrane protein, is necessary for complete activation of FGFR4 signaling [70].

FGFR4 is a trans-membrane receptor with an extracellular region consisting of three immunoglobulin-like domains, a hydrophobic trans-membrane region, and two intracellular regions with TK activity. When FGF19 binds to extracellular region of FGFR4, in the presence of its co-activator KLB, the intracellular region of FGFR4 is auto-phosphorylated and activated. Activation of FGFR4 causes phosphorylation of different adaptor proteins involved in the RAS/RAF/MEK/ERK, and PI3K/AKT signaling pathways (Figure 1). FGFR4 regulate epithelial-to-mesenchymal transition (EMT) by modulating the glycogen synthase kinase 3β (GSK3β)/β-catenin pathway and expression of E-cadherin, a key epithelial cell adhesion protein (Figure 1).

Aberrant activation of FGF19/FGFR4 signaling has been observed in many different human malignancies, including HCC [71, 72]. Multiple lines of evidence support the hypothesis that over-activation of FGF19/FGFR4 pathway, as well as *FGF19, FGFR4* and *KLB* gene amplifications, may promote HCC growth, malignant progression, metastasis and drug resistance [73–76]. Miura et al. [75] demonstrated that FGF19 was highly expressed in HCC tissues compared to normal liver tissues and that its expression correlated with tumor progression and poor prognosis. Recently, it has been proposed that FGF19 and KLB are potential biomarkers for prediction of early tumor recurrence in patients with resectable HCC [60]. Manipulation of *FGF19* gene expression resulted in different HCC cell responses to sorafenib treatment. FGF19 overexpressing-HCC cells displayed the lowest sensitivity to sorafenib treatment while, small interfering RNA (siRNA)-mediated FGF19 knockdown significantly increased drug sensitivity [75].

Given the potential oncogenic role of FGF19/FGFR4 pathway, the search for selective FGFR4 inhibitors has intensified. Some of these inhibitors are currently in phase I/II clinical trials (Table 1). Ponatinib (Iclusig®, Ariad Pharmaceuticals) is a third-generation FGFR4 inhibitor. It was approved in 2012 by the US FDA for treatment of two rare types of leukaemia. Ponatinib synergizes with sorafenib and significantly reduces HCC cell viability [75]. Different clinical studies are ongoing using both pan-FGFR inhibitors, such as erdafinib (JNJ-42756493; Balversa ®, Janssen Pharmaceutical) [77], and selective FGFR4 inhibitors, such as BLU-554 (Blueprint Medicines) and BLU-9931 [78]. In a phase I study, the pharmacokinetics and safety of erdafinib were evaluated in different cohorts of patients with solid tumor including: breast, lung, gastric, head and neck cancer, cholangiocarcinoma and lymphoma. Initially, patients (n = 193) with unresectable tumors were enrolled and treated with increasing doses of the drug to determine the well-tolerated dose. Erdafinib displayed acceptable toxicity with hyperphosphatemia being the most common side effect. Subsequently, a phase I/II study (NCT02421185) was performed and recently completed to evaluate the safety and pharmacokinetics properties of erdafinib in fifty-two Asian participants with advanced HCC. The results of this trial are currently unknown.

BLU-9931 is a potent irreversible and selective FGFR4 inhibitor developed to treat patients with advanced HCC with aberrant activation of FGFR4 signaling. This compound bound to Cys552, which is localized in the hinge region of FGFR4 and not found in the other FGFRs. In a preclinical study, Hagel et al. [79] demonstrated that BLU-9931 has potent dose-dependent antitumor effects on cancer cell lines exhibiting alterations in FGFR4 pathway. BLU-9931 strongly inhibited phosphorylation of FGFR4 downstream targets, such as p-ERK1/2 and p-AKT, and its efficacy was dependent on the expression of fully functional expression of FGF19, KLB and FGFR4 complex [79].

BLU-554 is another potent FGFR4-selective inhibitor that has been tested in a phase I dose-escalation/dose-expansion study in advanced HCC [80]. As stated previously, there are multiple FGFs. Recently it was shown that FGF19 acts as a driver mutation in HCC in certain patients. Selective patient screening was performed to identify HCC patients which might be selective to BLU-554 which targets the FGF19 signaling pathway. These patients were identified by immunohistochemistry (IHC) for aberrant FGF19 activation. Recently, a phase I first-in-human trial was performed to determine safety, pharmacokinetics (PK) and pharmacodynamics (PD). Fisogatinib (BLU-554), a small molecule kinase inhibitor, was determined to inhibit FGF19-positive growth of HCC [80]. Administration of fisogatinib was well tolerated with relatively minor side effects. The ORR was 17 % (11 out of 66 patients). As of December 2019, there was one complete response and ten partial responses. Three patients remained in response until the time of data cut off. The median duration of response in patients with FGF19 positive tumors was 5.3 months. The median PFS was 3.3 months. Additional studies with fisogatinib and FGF19 activation and survival of HCC patients are underway. Interesting and relevant for interpretation of these results, the authors observed that there was no beneficial response in thirty-two patients who were FGF19-negative. Additional combinational approaches may also be pursued.

In addition, BLU-554 is also being tested in phase I study (NCT02508467) in patients with advanced HCC to evaluate safety, PK and PD of the drug. This study is ongoing.

Recently, Joshi et al. [81] synthetized another potent selective FGFR4 inhibitor named H3B-6527, which covalently binds to Cys552 present in the ATP-binding domain of FGFR4 but not in the other FGFRs. This compound exhibited selective potent inhibitory effects on FGFR4 signaling in HCC cells over-expressing FGF19, due to *FGF19* gene amplification, and in a murine xenograft model [81]. H3B-6527 is currently being tested in a phase I study (NCT02834780) to evaluate tolerability, safety, pharmacokinetics and pharmacodynamics of the drug in patients with advanced HCC.

## Background and strategies based on targeting RAS/RAF/MEK/ERK pathway

The RAS/RAF/MEK/ERK pathway is also frequently dysregulated in human cancer due to mutations in upstream receptors molecules which pass their proliferative signals through this pathway, as well as mutations in component genes of the pathway, and regulatory molecules which normally serve to harness the pathway (e.g., phosphatases). The biochemical aspects of this pathway as well as the targeting of this pathway have been reviewed [82, 83]. This pathway is also frequently associated with the drug resistance of various cancers, including HCC [84, 85]. The multi-kinase inhibitor sorafenib was originally proposed to be a RAF inhibitor and as described previously it has been used to treat HCC patients [22, 86].

There have been multiple clinical trials with HCC patients and inhibitors that target RAF/MEK/ERK signaling. The effects of sorafenib on younger HCC patients is currently being examined in a phase II clinical trial (NCT01502410). The effects of the novel multikinase inhibitor donafenib have been examined in a clinical study (NCT02229071) with advanced HCC patients [87]. The effects of combination of sorafenib and tegafur/uracil (UFUR) have been examined in a phase II clinical trial (NCT00464919). This study correlated the ability of dynamic contrast-enhanced magnetic resonance imaging (DCE-MRI) to measure the vascular response with the clinical outcome.

The pharmacokinetic parameter K(trans) parameter, as measured by DCE-MRI, was determined to correlate well with tumor response and survival in HCC patients who underwent sorafenib and UFUR treatment [88]. The effects of combined RAF (sorafenib) and MEK1 (AZD6244) inhibitors on HCC patients have been examined in a phase Ib clinical trial (NCT01029418). A problem with treatment of various cancers with certain chemotherapeutic drugs or signal transduction inhibitors is the induction of MEK1. This trial demonstrated that the maximum tolerated dose (MTD) of the AZD6244 inhibitor was 75 mg daily when combined with 400 mg sorafenib twice a day in HCC patients. The authors stated that acceptable adverse events were observed [89]. Thus, it may be appropriate to treat certain patients with inhibitors that target two different signaling molecules in the same pathway. This may eliminate potential feedback loops (positive and negative) within the pathway.

A phase I clinical trial (NCT01668017) with the MEK1 inhibitor pimasertib was performed in Japan but was terminated by the sponsors. A phase II clinical trial (NCT01915589) examining the effects of the BAY86-9766 MEK inhibitor (refametinib) on HCC patients with mutant *RAS* has been performed. No results appear to have been posted as of December 2019. A phase I safety study was performed on combining refametinib and sorafenib and demonstrated acceptable safety profiles [90]. Therefore, a phase II clinical trial (NCT01204177) to examine the effects of combining the drugs refametinib and sorafenib was performed with HCC patients [91]. Interestingly, the best responses in this trial were observed with patients having *RAS* mutations. In this study, dose modifications were necessary to avoid side effects in most patients, however, antitumor activity was observed. The side effects included: aspartate aminotransferase elevation, diarrhea, nausea, rashes, and vomiting.

An additional phase II clinical trial (NCT01915602) examining the effect of combining refametinib and sorafenib in HCC patients with mutant RAS was performed. This trial was completed, but no results have been posted.

A phase II clinical trial (NCT02042443) with HCC patients with the MEK1 inhibitor trametinib (Mekinist®, GlaxoSmithKline; GSK1120212) has been performed. A phase I clinical trial (NCT02292173) examining the effects of treating HCC patients with trametinib and sorafenib was completed recently in 2019. Therefore, it is clear that there are numerous clinical trials examining the effects of combining the multi-kinase inhibitor sorafenib and various MEK inhibitors.

## Background and strategies based on targeting PI3K/AKT/mTOR pathway

The PI3K/AKT/mTOR pathway is another signaling pathway that is often deregulated in human cancer due to mutations/amplifications of upstream growth factor receptors, gene mutations in intrinsic pathway component, or mutations in phosphatases which serve to regulate the pathway (e.g., PTEN) [92–95].

The effects of the dual PI3K/bromodomain 4 (BRD) inhibitor SF1126 and the immune checkpoint inhibitor PD-1 MoAb nivolumab are being examined in the phase I clinical trial (NCT03059147) with HCC patients. This trial is still active, but not recruiting patients. No results of this trial appear to be available yet. The BRD inhibitor suppresses the expression of certain MYC-mediated factors. Nivolumab blocks the binding of PD-1 to its ligand. Nivolumab is approved for treatment of HCC. NCT02465060 (The MATCH Screening Trial) is a phase II clinical trial which will examine the effects of combinations of various inhibitors, including the PI3K-β inhibitor (GSK2636771) in HCC and other cancers. This trial is still recruiting patients. The purpose of the MATCH trial with multiple inhibitors as well as numerous cancer types is to determine, after initial standard anti-cancer therapy, how effective additional treatments based on genetic testing (genomics) is in patients that have progressed.

AKT lies downstream of PI3K and is a key molecule in the PI3K/PTEN/AKT/mTOR signaling pathway that is frequently dysregulated in various cancers, including HCC. There have been some clinical trials with AKT inhibitors. The AKT inhibitor MK2206 was in a clinical trial (NCT01239355) with HCC patients, however, that trial was discontinued due to discouraging results.

There have been at least thirty-four clinical trials with mTOR blockers/inhibitors. Everolimus (a.k.a. Rad001; Afinitor®, Novartis) has been evaluated in at least six clinical trials with HCC. The results of clinical trials with everolimus have been published in some cases. NCT00390195 was a phase I/II clinical trial with HCC patients. This study determined that the recommended daily dose of everolimus was 7.5 mg daily and that prophylactic anti-viral therapy should be provided to HBsAg-seropositive patients [96].

The effects of everolimus has been examined in at least one phase III clinical trial (NCT01035229) in HCC patients who failed on sorafenib treatment [97]. Unfortunately, this study did not reveal an increase in OS after everolimus treatment in advanced HCC patients either during sorafenib treatment or after failure on sorafenib.

The combination of everolimus and sorafenib has been evaluated in at least four additional clinical trials with HCC patients. The results of a phase II clinical trial (NCT01005199) which examined the effects of sorafenib with or without everolimus in advanced HCC patients have been published [98]. This trial revealed that combining 5 mg everolimus with full-dose sorafenib was possible, but it was more toxic than treatment with sorafenib by itself. Unfortunately, this study indicated that combining sorafenib with everolimus did not increase the efficacy of sorafenib by itself.

The combination of everolimus and estrogen receptor deprivation therapy has been evaluated in at least one phase II clinical trial (NCT01642186) with HCC patients. This trial is active, but not recruiting patients and no results appear to have been posted yet. The combination of everolimus and the anti-VEGF MoAb bevacizumab has been evaluated in at least one phase II clinical trial (NCT00775073) with HCC patients. This trial has been completed but results do not appear to have been published yet.

The mTORC1 blocker temsirolimus (a.k.a CCI-779; Torisel®, Wyeth Pharmaceuticals) has been examined in clinical trials with HCC patients. The combination of temsirolimus and sorafenib has been evaluated in at least one phase I clinical trial (NCT00775073) and one phase II (NCT01687673) with HCC patients. This trial is active, but not recruiting patients. No results appear to have been published yet.

The combination of temsirolimus and bevacizumab has been evaluated in at least one phase II clinical trial (NCT01010126) with HCC patients [99]. While some side effects were observed, the study indicated an overall response rate (ORR) of 19 % and OS of 14 months. The number of patients (n = 26) examined in this trial was relatively low. The authors suggested that drug concentrations need to be optimized. The combination of temsirolimus and lenalidomide has been evaluated in at least one phase I clinical trial (NCT01183663) with HCC patients.

The mTORC1 blocker rapamycin (a.k.a. sirolimus; Rapamune®, Wyeth Pharmaceuticals) has been examined in at least seven clinical trials with HCC patients. The combination of rapamycin and bevacizumab has been evaluated in at least one phase I clinical trial (NCT00467194) with HCC patients. This study demonstrated that phase II dose of rapamycin should be 4 mg when used in combination with bevacizumab. The authors indicated that this combination had promising clinical activity and anti-vascular activity was detected [100].

The combination of rapamycin and liver transplantation has been evaluated in at least three clinical trials (NCT00355862, phase III; NCT01374750, phase II; NCT03500848, phase II and III) with HCC patients. The phase III study demonstrated that rapamycin treatment after liver transplantation in HCC patients did not improve the long-term relapse-free survival (RFS) past 5 years. However, a benefit was observed in RFS and OS in rapamycin-treated liver transplant patients for the first three to five years. This benefit was observed more predominantly in the low-risk patients [101]. Finally, the dual mTORC1 and mTORC2 inhibitor CC-223 is being examined in at least one phase II trial (NCT03591965) with HBV positive HCC patients. This trial is currently recruiting HCC patients who had received one prior line of systemic therapy. Thus, there remains considerable interest in targeting the PI3K/AKT/mTORC1 pathway for treatment of HCC. Targeting may occur by a single inhibitor or a combined approach with either a kinase or immune checkpoint inhibitor.

## Background and strategies based on targeting TGFβ/TGFβR

TGFβs, activins, inhibins, nodal, growth and differentiation factors (GDFs), and bone morphogenetic proteins (BMPs), all belong to the TGFβ superfamily [102, 103]. In humans, thirty-three functional genes encode the TGFβ family polypeptides [104]. These polypeptides are composed of a signal peptide, required for secretion, a long pro-polypeptide, that, as a dimer, binds and activates the receptors. Initially TGFβs are synthesized and secreted as precursors that are processed by extra-cellular convertases to produce biologically active dimeric ligands [105].

In cancer, TGFβs have dual roles [106]. In early-stage tumors, the TGFβ pathway promotes cell cycle arrest and apoptosis [107–109] whereas, at advanced stages, the TGFβ pathway promotes tumor progression and metastasis by stimulating cancer cell motility, invasion, EMT, and cell stemness [107]. This functional switch is known as the “TGFβ paradox” [110]. TGFβ cytokines signal through a transmembrane receptor serine-threonine kinase complex (Figure 2). Two receptors have been identified: the Type I and Type II receptors. During activation, TGFβ first binds to the constitutively active Type II receptor (TβRII), which recruits and activates the TGFβ Type I receptor (TβRI). For some ligands, additional co-receptors are required for optimal ligand binding and activation of the type I-type II receptor heterodimer. The TβRI-TβRII complex starts the so-called canonical TGFβ signaling through C-terminal phosphorylation of the receptor-activated SMADs (R-SMADs), SMAD2 and SMAD3. Activated R-SMADs then form a complex with SMAD4 (Co-SMAD, common mediator SMAD) that shuttles to the nucleus where it can associate with other transcriptional co-factors at DNA elements of target genes, thereby regulating TGFβ target gene expression (Figure 2) [111, 112]. Together with SMAD-mediated canonical TGFβ signaling, the TGFβ receptors can also activate other intracellular pathways, referred as non-SMAD signaling pathways (Figure 2). The non-canonical (non-SMAD) TGFβ signaling pathways include: the PI3K/AKT/mTOR, RAS/RAF/MEK/ERK, p38^MAPK^ and JNK cascades, and pathways downstream of Rho-like GTPase signaling intermediates [113–115].

A large body of evidence indicates that TGFβ1 is an important cytokine that promotes tumor progression, as it induces EMT and activates the WNT pathway. In HCC patients, elevated plasma levels of TGFβ1 are correlated with shorter survival time [116]. TGFβ1 plays a pivotal role in processes such as fibrogenesis, angiogenesis, immunosuppression, and invasiveness. High concentrations of TGFβ1 are considered as potent negative prognostic markers in unresectable HCC patients [117]. Moreover, an inverse correlation between circulating TGFβ1 and E-cadherin levels has been reported in patients with HCC, a condition that recapitulates the EMT process [118, 119].

Different responses to TGFβ1 have been observed depending on the liver cell type. Thus, TGFβ triggers the activation of hepatic stellate cells into myofibroblasts, which start to produce extracellular matrix (ECM) components that initiate the fibrogenic process. In hepatocytes, TGFβ induces both cell death and EMT [120].

TGFβ induces EMT of malignant hepatocytes through stimulating cancer-associated fibroblasts (CAFs) proliferation. Activated CAFs modulate growth, intravasation and metastatic spread of HCC cells [121]. TGFβ activation of CAFs is related to down-regulation of E-cadherin and to the up-regulation of the SNAIL/PDGF signaling pathway [122–123]. Reduced expression of E-cadherin has been associated with poor HCC tumor prognosis and shorter disease-free survival [124]. TGFβ signaling was suppressed after treatment with the dual type I and type II TGFβR kinase inhibitor LY2109761, which restored E-cadherin expression and reduced the migration of HCC cells [125, 126]. A reduction of connective tissue growth factor (CTGF)-mediated cross talk between HCC cells and CAFs was observed after treatment with the inhibitor. Also decreased blood vessel formation occurred due the VEGF released from HCC cells [121, 127]. Recently, it was shown that LY2157299 (Galunisertib®, Eli Lilly), but not the D10 MoAb against TGFβRII, blocked both the canonical and non-canonical TGFβ pathways [128, 129].

Several studies have been started to evaluate safety and effectiveness of the TGFβRI kinase inhibitor galunisertib in patients with advanced HCC. A randomized phase II trial (NCT02178358), with the primary endpoint being evaluation of OS, of galunisertib in the presence and absence of sorafenib is ongoing.

A phase II clinical trial evaluating the MTD of galunisertib in combination with the anti-PD-1 MoAb (nivolumab) (NCT02423343) is in progress. A phase I trial galunisertib plus stereotactic body radiotherapy (SBRT) (NCT02906397) is also active, but not recruiting patients. Results of these studies have not been published yet.

A phase I study (NCT02947165), with the aim to characterize safety and tolerability as single agent of NIS793, a MoAb that specifically targets and binds to TGFβ, started in 2017. NIS793 is also being evaluated in combination with PDR001 (an anti-PD-1 MoAb) in patients with advanced malignancies, including HCC (NCT02947165).

## Background and strategies based on targeting Aurora kinase

Mitotic events are regulated by reversible protein phosphorylation events powered by specific protein kinases and phosphatases, among them the Aurora kinases (AURKs). Aurora kinases belong to a family of serine/threonine kinases consisting of three members: Aurora A (AURKA), Aurora B (AURKB) and Aurora C (AURKC). Aurora kinases are composed of an N-terminal domain (39-139 aa), a kinase domain (250-300 aa) and a C-terminal domain (15-20 aa). The C-terminal domain of the kinase domain display a conserved residue at Thr288 (AURKA), Thr232 (AURKB) and Thr195 (AURKC), which upon phosphorylation induces a conformational change essential for the kinase activity [130, 131]. In the N- and the C-terminal domains are degrons that regulate the degradation of Aurora proteins at the end of mitosis.

Therefore, AURKs play pivotal roles in cell division and duplication, despite this, or perhaps because of this, it was observed that both AURKA and AURKB are upregulated in most human solid tumors [132–134], including HCC [135]. Usually their altered expression levels in cancer is considered a poor prognosis factor [132–134]. In tumor cells, AURKA induces cell proliferation, survival and drug resistance through interacting with oncogenic pathways, such as MYC, PKC/RAF/MEK/ERK, BCR/ABL, NF-κB, Wnt/β-catenin or the PI3K/AKT pathways [136–138], modulating pro-apoptotic (BCL2, MCL1) and anti-apoptotic (BAX, BIM, PUMA, APAF) proteins [136]. In addition, AURKB is involved in tumor cell proliferation and survival regulating CDK1, TP53 and inhibiting caspase-3 expression [139, 140]. Therefore, AURKs have become attractive drug targets for cancer therapy [141, 142]. The Aurora kinase B, in particular, may be an appropriate anticancer target as its inhibition rapidly results in mitotic catastrophe followed by senescence [143, 144]. The mitotic catastrophe occurs via TP53-independent cell death, which is likely a consequence of premature or inappropriate entry into mitosis [145].

Currently, over a dozen AURKs inhibitors have entered clinical trials [141]. Some are Aurora sub-type selective, i.e. AURKA selective, such as MLN8054, MLN8237, VX-689/MK-5108 and ENMD 2076; or AURKB selective, such as AZD1152 and GSK1070916. Other inhibitors are pan-selective, AURKA and AURKB selective, such as VX-680, PHA-739358, CYC116, SNS-314, PF3814735, AT-9283, R-763/AS-703569, AMG 900 and KW-2449 [141].

In human HCCs, AURKA and AURKB are overexpressed and are associated with aggressiveness, early recurrence and poor prognosis [145, 146]. Most of antitumor studies on AURKs inhibitors in HCC are still in preclinical phase.

The AURKA inhibitor, MLN8237 (Alisertib®, developed by Takeda) is a new reversible oral small-molecule selective inhibitor. MLN8237 inhibits cell viability in dose-dependent manner and strongly synergizes with sorafenib in inhibition of HCC progression, by inducing cell cycle arrest and apoptosis. These drug combinations affects also migration, invasion, through inhibition of p-AKT and p-p38^MAPK^ and their downstream genes, such as *VEGFA*, *cyclin D1 (CCND1)* and *cyclin-dependent kinase 4* (CDK4) [147]. These results suggest that the MLN8237 and sorafenib combination may be a novel therapeutic approach for HCC treatment.

The pan-Aurora kinase inhibitor PHA-739358 (Danusertib®, Nerviano Medical Sciences), is an AURKA/B/C inhibitor, which has been tested in several phase II trials in solid and haematological tumors. In HCC cells, it inhibits cell proliferation and induces autophagy through the PI3K/AKT/mTOR signaling pathway [148].

The AURKA inhibitor VE-465 suppressed proliferation, histone H3 (Ser10) dephosphorylation, events involved in mitosis and apoptosis in HCC cells. Treatment with VE-465 induced apoptosis and inhibited tumor formation in a human HCC xenograft model [149]. These results suggest that AURKA is a promising antitumor target, and that AURKA inhibitor may be a valuable agent against HCC.

PHA-739358 is a novel pan-selective AURKA inhibitor. PHA-739358 completely suppressed HCC cell proliferation *in vitro* and inhibited HCC growth *in vivo* in an animal model. In addition, combination of PHA-739358 with sorafenib resulted in an additive effect on tumor growth inhibition [150], thus highlighting that inhibition of AURKA, either alone, or in combination with sorafenib, may be a promising therapeutic approach for HCC. Currently PHA-739358 is under evaluation in a phase II clinical trial in patients with different types of solid tumors, however, this trial does not include HCC.

A selective inhibitor of AURKB, AZD1152 (Barasertib®, AstraZeneca) acts by suppressing histone H3 phosphorylation, resulting in accumulation of aneuploid (4N) cells and cell death [151]. Furthermore, treatment with AZD1152 significantly inhibited tumor growth of subcutaneous human HCC xenografts, as well as decelerated tumor growth and increased survival in an orthotopic HCC model [151]. These results suggested that AZD1152 could be a promising drug for the treatment of HCC.

The kinase inhibitor R1498 targets multiple Aurora kinases and other proteins, including AURKA, AURKB and VEGFR2, and affects both angiogenic and mitotic pathways. The *in vivo* antitumor efficacy of R1498 was tested in human cancer xenograft models using a panel of gastric cancer and HCC cell lines. R1498 treatment displayed growth inhibition and tumor regression [152].

Chiauranib is an AURK inhibitor in a clinical trial in HCC (Figure 3). Chiauranib is a novel orally active multi-target inhibitor that simultaneously inhibits the angiogenesis-related kinases (VEGFR2, VEGFR1, VEGFR3 and PDGFRα), as well as kinases involved in stimulating cell proliferation, such as c-KIT and colony stimulating factor-1R (CSF-1R). Chiauranib is in a phase I clinical trial with HCC patients (NCT03245190). This clinical trial is in the recruiting stage.

## Background and strategies based on targeting CDKs

HCC can develop from uncontrolled cellular proliferation that results from disruption of normal cell cycle regulatory checkpoints [153, 154]. The cyclin-dependent kinases (CDKs) are serine/threonine kinases proteins that control cell cycle progression (Figure 3). CDK activity depends on their association with non-catalytic regulatory subunits, referred as cyclins. There are four essential CDKs required for cell cycle progression: CDK1, CDK2, CDK4, and CDK6. Each CDK is associated with a regulatory cyclin subunit. Activation of the respective CDK determines cell cycle progression starting from the resting state (G_0_), to the growth phase (G_1_), through DNA replication (S), and finally to cell division phase (M). Cells enter the G_1_ phase after stimulation with mitogenic signals, with the intracellular increase of D-type cyclins (D1, D2 and D3), resulting in cyclin D/CDK4 and cyclin D/CDK6 complexes [154, 155]. This complex determines the phosphorylation and inactivation of the retinoblastoma (RB) protein, which leads to the release of E2F transcription factors, which induce expression of cyclin E. Cyclin E binds CDK2 and causes the transition into S phase. The G_1_/S transition is a critical point of the cell cycle progression. The S phase is characterized by DNA replication. Cyclin A binds CDK2 to drive the cell cycle from the S phase to G_2_. CDK1/cyclin B complex controls the transition into the G2/M phase. Finally, the complex CDK3/cyclin C regulates exit from the cell cycle at G_0_ phase (Figure 3) [156, 157].

CDK overexpression is often observed in HCC, which can result from inactivation of CDK inhibitory proteins, such as p16^Ink4^, p21^WAF1/CIP1^ and p27^KIP1^ [153, 154, 158]. Therefore, CDK inhibitors constitute an attractive therapeutic option for HCC treatment [154, 159].

CDK1 expression is upregulated in liver samples from HCC patients in comparison to non-tumor tissues [160]. Moreover, high levels of CDK1 expression is predictive of tumor recurrence [160].

Various CDK1 inhibitors are being evaluated, including P276-00 (Riviciclib®, Piramai Enterprises Ltd.) and flavopiridol (Alvocidib®, Tolero Pharmaceuticals, Inc.). Interestingly, synergistic effects on the induction of apoptosis upon combination of alvocidib and doxorubicin were observed in an *in vivo* HCC model [161]. In the clinical setting of a phase II trial (NCT00087282), the combination of alvocidib and irinotecan was evaluated in patients with advanced HCC. Sequential irinotecan and alvocidib administration did not appear to have clinically relevant antineoplastic activity. Ten patients were evaluable for response: one had stable disease (SD) >1 year and nine had disease progression.

CDK2 regulates the G1-S phase by binding cyclin E and cyclin A. The CDK2-cyclin E complex contributes to the uncontrolled growth of HCC [162–164]. The complex is endowed with catalytic activity, which determines the phosphorylation of the E2F2 factor that is necessary for termination of S phase. CDK2 was reported to be hyperactive in 80% of the cases of HCC [165]. Xylocydine, a CDK2 specific inhibitor that selectively down-regulates CDK2 activity, had significant growth inhibitory effects in HCC cells *in vitro*, as well as suppressed tumor growth *in vivo* in murine xenografts [166].

CDK4 is another kinase that is expressed at high levels in HCC. The levels of CDK4 mRNA and protein were analysed in fifty-nine pairs of HCCs and adjacent normal tissues [167]. CDK4 was upregulated in 73% of the HCC samples and expression of CDK4 correlated with tumor size and stage [167]. Moreover, gene expression profiles revealed overexpression of CDK4 mRNA in HCC tissues [168]. CDK4 is a potential prognostic marker for HCC [167, 168].

Given the importance of CDK4 in liver cancer, CDK4 inhibitors are utilized in HCC treatment. Palbociclib (PD-0332991; Ibrance®, Pfizer), is a reversible, selective CDK4/6 inhibitor. Palbociclib has been recently approved by the US FDA for treatment of patients with breast cancer [169]. In HCC cell lines, palbociclib promotes a reversible cell cycle arrest and the induction of cellular senescence, alone or in association with sorafenib [170] and enhances radiosensitivity [171]. A phase II clinical trial (NCT01356628) testing palbociclib in HCC patients is underway as a second-line therapy after sorafenib failure.

An atypical CDKs member is CDK5, this protein does not participate in cell cycle progression and is not activated by cyclins. CDK5 is indispensable for normal brain development, neuronal survival and synaptic plasticity [172–174]. In HCC, CDK5 is highly expressed in tumor tissues, regulates DNA damage response, and promotes angiogenesis through interactions with inducible hypoxia factor 1α (HIF-1α) [175, 176].

Combination analysis of immunohistochemistry (IHC) with high-throughput RNA sequencing (RNAseq), and microarray data from The Cancer Genome Atlas (TCGA), confirmed the relationship between CDK5 levels and progression of HCC [177]. Furthermore, the same authors demonstrated that CDK5 knockdown (KD) by siRNA inhibited cell growth and induced apoptosis *in vitro* [177]. Combining sorafenib and CDK5 inhibition, either by genetic KD by short hairpin RNA (shRNA), or pharmacologic inhibition with dinaciclib (a.k.a SCH-727965), synergistically compromised HCC progression *in vitro* or in an *in vivo* animal model [178].

## Background and strategies based on targeting HDACs

Histone acetylation and deacetylation are epigenetic regulatory mechanisms that play critical roles in the modulation of chromatin and the regulation of gene expression [179]. Changes in acetylation/deacetylation patterns regulate transcription [180]. These changes may be due to altered expression or mutation of genes that encode histone acetyltransferase (HAT), histone deacetylase (HDAC) enzymes, or their binding partners. These events may contribute in part to carcinogenesis [181].

There are multiple HDAC classes with different functional characteristics and with specific cellular localization: class I (HDAC1, HDAC2, HDAC3, HDAC8), class IIa (HDAC4, HDAC5, HDAC7, HDAC9), class IIb (HDAC6, HDAC10), and class IV (HDAC11). There is also a class III of HDACs, which are named sirtuins (SIRT1, SIRT2, SIRT3, SIRT4, SIRT5, SIRT6, SIRT7) (Figure 4).

Aberrant regulation of HDACs can lead to initiation and progression of HCC [182]. Gene expression analysis revealed increased expression of *HDAC1*, *HDAC2* and *HDAC3* in HCC tissues with respect to non-tumor areas. HDAC overexpression correlated with tumor dedifferentiation and proliferative activity [183–185]. In a cohort of 334 human HCCs, aberrant expression of several HDACs, and increased copy numbers of the *HDAC3* and *HDAC5* genes were observed [186].

In several studies, sirtuins serve as useful prognostic biomarkers in HCC [187]. Overexpression of SIRT1, SIRT2 and SIRT7 was linked to increased expression of oncogenic cell cycle genes. SIRT1 levels are elevated in HCC tissues in comparison to non-malignant tissues [188]. Contrasting results on the role of SIRT6 in HCC have been reported. It was shown that downregulation of SIRT6 induced hepatocarcinogenesis and inhibited apoptosis [189], while another study revealed that SIRT6 overexpression inhibited apoptosis [190]. Thus, HDAC inhibitors (HDACi) represent a promising therapy for HCC treatment, either as monotherapy or in combination with other anticancer drugs.

Trichostatin A (TSA) is a hydroxamate HDCAi. TSA inhibits the growth of hepatic tumors through cell cycle blocks and activation of apoptosis [191]. Recently, Chen et al. [192] have shown that the effects of TSA on HCC cells may be increased by pre-treatment with sorafenib, via the inhibition of RAF/MEK/ERK and NF-κB signaling pathways. Furthermore, TSA increases the killing of HCC cells indirectly by increasing natural killer (NK) activities, through increasing the expression of NKGD2 ligands (MICA/B and ULBP1/2/3) and directly increasing apoptosis [193].

Another hydroxamate HDACi exhibiting preclinical antitumor activity in HCC is vorinostat (suberoylanilide hydroxamic acid, SAHA; Zolinza ®, Merck Sharp & Dohme Ltd). Vorinostat, blocks proliferation of HCC cells and activates cell death through apoptosis [194] and autophagy [195]. Vorinostat inhibits HIF-1α, which results in inhibition of angiogenesis [196] and upregulation of microRNAs (miRs), which act as tumor suppressors [197]. Co-treatment with vorinostat and oxaliplatin exhibited synergistic anticancer effects in HCC cells *in vitro* and in an *in vivo* in animal model [198]. A phase study I (NCT01075113) is currently investigating the side effects and the best doses of vorinostat when given together with sorafenib in patients with advanced HCC.

Panobinostat (LBH589; Farydak®, Novartis), is a new hydroxamic acid-derived HDACi with promising anticancer effects. In 2016, the FDA approved panobinostat for treatment of patients with multiple myeloma. Panobinostat activates alternative apoptotic pathways in HCC cells, also in TP53-deficient cells, and reduced angiogenesis in tumor xenografts [199], through modulation of extracellular signaling cascades via a CTGF-dependent pathway [200]. Moreover, panobinostat in combination with sorafenib led to strong antitumor effects *in vitro* and *in vivo,* through the activation of apoptosis and autophagy, and inhibition of vessel density and tumor volume [186]. Combination of panobinostat with sorafenib has been evaluated in HCC patients in two phase I clinical trials (NCT00873002, NCT00823290). Clinical trial NCT00873002 was terminated due to severe dose-limiting toxicity, whereas results of the NCT00823290 study are not available yet.

Resminostat (4SC-201, RAS2410) is an inhibitor for class I HDACs. This HDAC induced cell death in HCC cells, and co-treatment with sorafenib had synergistic effects in mesenchymal HCC cells, which were resistant to sorafenib-induced apoptosis [201]. In the SHELTER study (NCT00943449) the therapeutic combination of sorafenib and resminostat prolonged survival in patients with advanced HCC. In this study, efficacy, evaluated as PFS was 12.5% for resminostat and 62.5% for resminostat plus sorafenib. Median TTP and OS were 1.8 and 4.1 months for resminostat and 6.5 and 8.0 months for the combination, respectively [202]. In contrast, in a phase I/II follow-up study, this therapeutic combination evaluated in Asian HCC patients did not reveal benefits in OS (NCT02400788).

Belinostat (PXD101; Beleodaq®, TopoTarget) is a hydroxamate HDACi that in 2014 was approved by the FDA for treatment of patients with peripheral T-cell lymphoma (PTCL). Preclinical data obtained in HCC cells, revealed that belinostat inhibited cell growth and induced apoptosis [203]. In addition, a synergistic effect was observed in HCC cells after combining belinostat with the proteasome inhibitor bortezomib [204]. Recently, it was shown that combination of belinostat with ICIs enhanced its antitumor efficacy in a murine HCC model [205]. However, in a phase I/II trial (NCT00321594) while belinostat was well tolerated in patients with unresectable HCC, no efficacy was reported [206].

## IMMUNOTHERAPY IN HCC

The development and progression of a tumor depends on evasion of immunological surveillance. Although the immune tolerance mechanisms are complex and not completely understood, nevertheless they have provided the main rationales for the development of immunotherapy as an effective therapeutic strategy in cancer, including HCC treatment [207]. These mechanisms include dysfunction of effector T-cells, defects in antigen presentation, alterations in immune checkpoint molecules and aberrant cytokine profiles.

Immune checkpoint proteins are defined as surface glycoproteins that send inhibitory signals for immune cells, mainly T cells or natural killer (NK) cells, preventing their activation. The immune checkpoints are therefore fundamental for the induction and maintenance of the immune tolerance of tumors. Under physiologic conditions, these molecules resolve T-cell activation during immune responses in such a way as to limit collateral tissue damage.

The two most widely studied immune checkpoints to date in human cancer are the PD-1 (a.k.a. CD279) and the CTLA-4 (a.k.a. CD152), which were discovered in 1992 and 1995, respectively [208, 209].

These two immune checkpoints are known to control different phases and signaling processes of the immunological surveillance. In the initial phase of "priming" of naïve T cell activation, the binding of the CTLA-4 to its receptor inhibits the stimulatory signals, and blocks the development of potentially self-reactive T cells [210]. In the next "effector" phase of the immune response, the interaction of PD-1 with its ligand, PD-L1, plays an important role, leading to the regulation of cytotoxic T lymphocytes (CTLs) that were previously activated during the priming phase [211]. However, there are many other proteins, such as lymphocyte activation gene-3 (LAG-3, CD223), B and T cell lymphocyte attenuator (BTLA, CD272), T cell immunoglobulin-3 (TIM-3), etc., which play important roles in the immune response to cancer, although their mechanisms of action remain unclear [212].

## Background and strategies based on targeting CTLA-4

CTLA-4 is a type 1 transmembrane glycoprotein of the CD28-B7 immunoglobin superfamily, and is found as a homodimer of 41-43 kDa on the cell surface of activated T cells, regulatory T cells (Tregs), and naïve T cells [213]. CTLA-4 is an intracellular protein in resting T cells, and it translocates to the cell surface after a costimulatory signal through CD28 and T cell receptor (TCR) engagement. At the cell surface, CTLA-4 competes with CD28 for binding to its ligands such as the B7 molecules B7.1 (a.k.a. CD80) and B7.2 (CD86) expressed on the surface of an antigen-presenting cell (APC) (Figure 5).

CTLA-4 binds to CD80 and CD86 with greater affinity/avidity than CD28. Since the cytoplasmic tail of CTLA-4 has no intrinsic enzymatic activity, the delivery of negative signal is probably due to its ability to associate with different signaling molecules, including the serine/threonine phosphatase PP2A (PP2A) and the SH2 domain-containing tyrosine phosphatase-2 (SHP-2) through association with the YVKM motif (Figure 5).

After binding to its ligands, CTLA-4 delivers inhibitory signaling into the T cell, with subsequent arrest of both proliferation and activation. Therefore, CTLA-4 blockade can lead to the removal of this molecular "brake" and consequently restore the activation of T cells.

In 2000, two CTLA-4–blocking MoAbs, ipilimumab and tremelimumab, entered clinical trials for treatment of patients with cancer. Currently, only ipilimumab (MDX-010; Yervoy^®^, Bristol-Myers Squibb) has been approved by FDA for cancer treatment.

Ipilimumab is a fully humanized IgG1 MoAb that was initially approved in 2011 as monotherapy for treatment of patients with advanced melanoma [214, 215]. It is under investigation in a phase II trial (NCT03222076), in HCC patients, which are resectable in the context of pre-surgical therapy.

Tremelimumab (CP-675, 206; Astra-Zeneca) is a fully humanized IgG2 MoAb. It has been evaluated in numerous phase III trials for treatment of several cancer types, but it has not yet received FDA approval. A small phase II trial (NCT01008358) of 21 HCC patients with underlying chronic HCV infection was conducted in 2013 [216]. Although this study demonstrated antiviral effects, associated with an enhanced specific anti-HCV immune response, and antitumor activity, associated with an ORR of 17.6%, a disease control rate (DCR) of 76.4%, and median TTP of 6.5 months, concerns were raised about the toxicity associated with this monotherapy compared to anti-PD-1 therapy. However, the favorable antitumor efficacy of tremelimumab opened new opportunities for its use as monotherapy (NCT02519348), in combination therapies with other immune checkpoint inhibitors (ICIs) (NCT03298451; NCT02519348), or with loco-regional therapies (LRT) (NCT01853618).

## Background and strategies based on targeting PD-1 and PD-L1

PD-1 is a monomeric transmembrane protein of 50-55-kDa, structurally related to CTLA-4 and CD28, with immunoglobulin-like extracellular domains, a transmembrane domain and a cytoplasmic tail containing two tyrosine-based signaling motifs, i.e. an immunoreceptor tyrosine-based inhibitory motif (ITIM) and an immunoreceptor tyrosine-based switch motif (ITSM) (Figure 6) [217]. These two cytoplasmic motifs are involved in PD-1-mediated immunosuppressive effects. PD-1 is expressed at the cell surface of activated T cells, B cells, NK cells, Tregs, monocytes, and dendritic cells (DCs) [218].

The ligands of PD-1 (PD-L1 and PD-L2) belong to the B7 members. PD-L1 (a.k.a. B7-H1) and PDL-2 (B7-DC) are both type I transmembrane proteins. PD-L1 shows broader cell and tissue distribution. PD-L1 is constitutively expressed by T cells, B cells, macrophages and DCs, and its expression is increased following activation of these cells. Expression of PD-L2 is limited to professional APCs. PD-L1 and PD-L2 are also expressed in different tumor types, including HCC where their expression correlates with survival and tumor recurrence [219–223].

The binding of PD-1 with its ligands, PD-L1 or PD-L2, inhibits activation of CD8-positive T-cell by blocking TCR- and CD28-mediated signaling pathways [224]. Upon interactions between PD-1 and either PD-L1 or PD-L2, SHP2 is recruited to the ITSM domain of the PD-1 cytoplasmic tail, leading to inhibition of PI3K/AKT signaling, which in turn leads to arrest of T cell proliferation and activation (Figure 6), achieving a status known as of T cells exhaustion [225]. Tumor cells, which express PD-L1 and PD-L2 molecules at their surface, use this mechanism to avoid immune surveillance and to survive via activation of antiapoptotic signals mediated by PD-L1. Therefore, blockade of PD-1/PD-L1 (or PD-1/PD-L2), by anti-PD-1 or anti-PD-L1 (anti-PD-L2) can lead to growth inhibition and restoration of T cells activation.

Several anti-PD-1 antibodies have been developed (Table 2). Nivolumab (Opvido®, Brystol-Myers Squibb) is a fully humanized MoAb. Based on a phase I/II trial (CheckMate 040; NCT 01658878), in 2017, nivolumab received accelerated FDA approval for treatment of patients with advanced HCC who had previously received sorafenib [226]. This study revealed an ORRs of approximately 20% in all patients, irrespective of etiology (presence or absence of HBV or HCV infection), and an ORR of 23% and OS rate of 82% at 9 months in naive patients, thus supporting the evaluation of nivolumab as a first-line therapy for patients with advanced HCC [226]. Although the expression of PD-1 and PD-L1 on tumor-infiltrating lymphocytes was not evaluated in this study, objective responses occurred regardless of PD-L1 expression on tumor cells. The positive results of this phase I/II trial stimulated the launch of an open-label phase III randomized trial of nivolumab, in a first-line setting, versus sorafenib for comparisons of PFS and OS (CheckMate 059; NCT02576509). This study is ongoing.

**Table 2:** ICIs under clinical evaluation in HCC (as of December 2019).

**Targets**	**Agent(s) (trade name; code name; Company)**	**Phase**	**Status**	**ClinicalTrials.gov Identifier**
***Single agent***				
**CTLA-4**	Tremelimumab	II	recruiting	NCT02519348
	Ipilimumab (Yervoy^®^, MDX-010, Bristol-Myers Squibb)	II	recruiting	NCT03222076
**PD-1**	Nivolumab (Opvido®; Brystol-Myers Squibb)	III	active, not recruiting	NCT02576509
		III	recruiting	NCT03383458
	Pembrolizumab (Keytruda®; MK-3475; Merck Sharp and Dohme)	I II	recruiting active, not recruiting	NCT02595866 NCT02702414
		II	recruiting	NCT03419481
		II	recruiting	NCT03163992
		III	recruiting	NCT03062358
		III	active, not recruiting	NCT02702401
	Tislelizumab (BGB-A317; BeiGene)	II	active, not recruiting	NCT03419897
	Camrelizumab (SHR-1210, HR-301210; Jiangsu Hengrui Medicine/Incyte)	II	active, not recruiting	NCT02989922
**PD-L1**	Durvalumab (Imfinzi®; MEDI4736; Astra Zeneca/MedImmune)	III II	active, not recruiting active, not recruiting	NCT03298451 NCT03389126
	Avelumab (Bavencio®; EMD Serono, Inc.)	I	active, not recruiting	NCT02699515
	MSB0011359C (M7824; Merck KGaA)			
***Combination of ICIs***				
**CTLA-4, PD-1**	Ipilimumab + Nivolumab	I/II	active, not recruiting	NCT01658878
		II	recruiting	NCT03222076
**CTLA-4, PD-L1**	Tremelimumab + Durvalumab	III	active, not recruiting	NCT03298451
		II	recruiting	NCT02519348
***Combination of ICI with targeted agents***				
**PD-1, TKIs**	Nivolumab + Sorafenib	I/II	active, not recruiting	NCT01658878
	Nivolumab + Cabozatinib	I/II	active, not recruiting	NCT01658878
	Nivolumab + Lenvatinib	I	active, not recruiting	NCT03418922
	Pembrolizumab + Sorafenib	I/II	recruiting	NCT03211416
	Pembrolizumab + Lenvatinib	I/II	recruiting	NCT02501096
	Pembrolizumab + Regorafenib	I	recruiting	NCT03347292
	Spartalizumab (PDR001; Novartis) + Sorafenib	II	active, not recruiting	NCT02988440
**PD-L1, TKIs**	Atezolizumab + Cabozantinib (Cabometyx® , Exelixis, Inc.)	III	recruiting	NCT03755791
**PD-1, VEGF**	Nivolumab + Bevacizumab	I	active, not recruiting	NCT03382886
**PD-L1, VEGF**	Durvalumab + Bevacizumab	II	recruiting	NCT02519348
	Atezolizumab (Tecentriq®; Genentech) + Bevacizumab	III	recruiting	NCT03434379
**PD-L1, VEGF, KIT, PDGFR**	Avelumab + Axitinib	I	completed, no results posted	NCT03289533
***Combination of ICI with loco-regional therapy***				
**DEB-TACE, PD-1**	DEB-TACE + Nivolumab	II	recruiting	NCT03572582
**TACE, PD-1**	TACE + Pembrolizumab	I/II	recruiting	NCT03397654
**TACE, RFA, CTLA-4**	TACE + Tremelimumab; RFA + Tremelimumab	I	active, not recruiting	NCT01853618
**SBRT, CTLA-4, PD-1**	SBRT + Ipilimumab + Nivolumab	I	recruiting	NCT03203304
**DEB-TACE, CTLA-4, PD-L1**	DEB-TACE+ Tremelimumab + Durvalumab	II	recruiting	NCT03482102
**Radiation, CTLA-4, PD-L1**	Radaition, Tremelimumab + Durvalumab	II	recruitting	NCT03482102

adverse events (AEs); Treatment-emergent adverse events (TEAEs); Objective Response Rate (ORR); Transarterial chemoembolization (TACE); stereotactic body radiotherapy (SBRT); radiofrequency ablation (RFA); dose limiting toxicities (DLTs); Drug-eluting Bead Transarterial Chemoembolization (DEB-TACE); serious adverse events (SAEs)

Pembrolizumab (MK-3475; Keytruda®, Merck Sharp and Dohme) is a humanized mAb against human PD-1. It has been investigated in the KEYNOTE-224 phase II trial, as a second-line treatment in HCC patients previously treated with sorafenib [227]. In this study, results were similar to those obtained with nivolumab, with on ORR of 17% observed in all patients regardless of etiologies. Median PFS and OS were 4.9 months and 12.9 months, respectively. Pembrolizumab has been evaluated in two phase III trials as a second-line therapy in HCC patients who had progressed on, or were intolerant, to sorafenib (KEYNOTE-240, NCT02702401; KEYNOTE-394, NCT03062358). However, as revealed by press information on February 19, 2019, KEYNOTE-240 trial failed to reach its co-primary endpoints, i.e. PFS and OS [228], compared to placebo and best supportive care. The other randomized, double-blind phase III trial, KEYNOTE-394, is still recruiting Asian patients with advanced HCC, who were previously treated with sorafenib. This study is evaluating pembrolizumab in combination with best supportive care, versus placebo in combination with best supportive care. In addition, there are numerous ongoing trials examining pembrolizumab in combination with other treatments.

Clinical trials evaluating the effects other anti-PD-1 MoAbs, such as tislelizumab (BGB-A317; BeiGene) and camrelizumab (SHR-1210, HR-301210; Jiangsu Hengrui Medicine/Incyte) as single agents are also ongoing in HCC. In addition, other anti-PD-1 MoAbs, such as spartalizumab (PDR001; Novartis), are used in combination with other therapies.

Finally, several anti-PD-L1 antibodies have been developed (Table 2). Durvalumab (Imfinzi®, Astra Zeneca/MedImmune), a human MoAb to PD-L1, received accelerated approval by the FDA in May 2017 to treat patients with cancers in the bladder and urinary tract. In HCC, a phase I/II trial in a cohort of 40 patients with advanced stage of disease was completed [229]. The ORR of this study was 10.3%, the median OS was of 13.2 % and median PFS was 2.7 months. A phase III trial (NCT03298451) of durvalumab as first-line treatment in patients with advanced HCC is currently underway.

Avelumab (Bavencio®, EMD Serono, Inc.) is a fully humanized MoAb that was initially approved by the FDA in 2017 as first-line treatment for metastatic Merkel cell carcinoma (MCC). A phase II study (NCT03389126) is currently investigating avelumab as monotherapy in patients with advanced HCC, after prior sorafenib treatment.

## Combination of immune checkpoint inhibitors with other HCC treatments

Many ICIs are currently being investigated in combination with other ICIs, with kinase inhibitors, with molecular targeted agents, or with loco-regional therapies, such as TACE, radiofrequency ablation (RFA), and radiation (Table 2).

A phase II study (NCT02519348) evaluating safety of combination of two ICIs, durvalumab and tremelimumab, in forty patients with unresectable HCC, with or without associated HBV or HCV infection who progress on, are intolerant to, or have refused sorafenib therapy, has been conducted [230]. The study revealed an objective response rate of 40% in the 20 uninfected patients (no HBV or HCV) and of 25% in all forty patients [230]. No unexpected safety problems were observed in the patients included in this study. Thus, a larger phase III trial (NCT03298451) is currently recruiting participants.

Atezolizumab (Tecentriq®) is a PD-L1 blocking MoAb developed by Genentech, and has been approved by the FDA (May, 2016) for the treatment of bladder and urinary tract cancers (urothelial carcinoma), and subsequently (October, 2016) for the treatment of metastatic non-small cell lung cancer (NSCLC). A multicenter phase Ib trial (COSMIC-021; NCT03170960) evaluating atezolizumab in combination with TKI cabozantinib is currently recruiting patients with advanced HCC to determine the MTD and ORR (COSMIC-021; NCT03170960). This combination is also being studied in an ongoing multicentre phase III trial, (COSMIC-312; NCT03755791) which expects to recruit about 640 patients, with the aim to evaluate the potential in first-line therapy of cabozantinib and atezolizumab in comparison with sorafenib for patients with advanced HCC (Table 2). The primary endpoints for the study are the duration of PFS and OS.

Recently, the results from the phase Ib study of atezolizumab in combination with the anti-VEGF MoAb bevacizumab in sixty-eight patients with advanced HCC were reported at the ESMO 2018 Congress [231]. The ORR was 34% and PFS rate at 6 months was 71%, whereas median duration of response (DOR) and median OS have not yet been reached. Interestingly, responses were observed in all patient subgroups, including those with AFP ≥400 ng/ml. Due to the encouraging results, a phase III IMbrave150 trial (NCT03434379) is currently recruiting patients.

Loco-regional therapies are commonly used as primary treatments in patients with unresectable HCC. Now they are being evaluated in combination with ICIs in patients with advanced HCC. The rationale for this approach is based on the assumption that killing cancer cells with these therapies, promote the release into the blood of tumor-associated antigens and neoantigens. This results in the activation of immune response that can recognize and kill cancer cells that survived. Therefore, addition of ICIs could reinforce these effects. In particular, a phase II trial (NCT03572582) of combination of nivolumab with TACE has been initiated with the aim to evaluate ORR. Other clinical trials of combination of LRTs (RFA, TACE, radiation) with pembrolizumab (NCT03397654), or tremelimumab (NCT01853618), or ipilimumab + nivolumab (NCT03203304), or durvalumab + tremelimumab (NCT03482102), are active but not recruiting or are still recruiting patients, most of them should be completed in 2020-2021.

## THERAPY IN ELDERLY HCC PATIENTS

The definition of elderly patient is traditionally of a subject aged >65 years. However, a real cut off point cannot be defined as the aging process is an individual process so, according to the definition of the World Health Organization (WHO), it is necessary to talk about Healthy Aging and the age above which this concept is most frequent is >60 years. Nevertheless, most of the clinical studies on HCC classify the population as elder if 75 years old and extremely old if over 80 years [232–234].

For various socio-economic reasons, life expectancy in the last 20 years has increased all over the world when compared to 1900. According to WHO in Europe there has been an increase from 76.7 years in 2010 to 77.9 years in 2015, and Italy ranks second in Europe and eighth in the world as regard the life expectancy being 82.7 years in all-population, divided in males 80.5 and in females 85 years [235]. With the increase in life expectancy there is also an increase in the frequency and incidence of degenerative diseases, in particular of neoplasms, which in the age group > 65 years have an incidence 11-fold higher than in the younger population [236].

International guidelines suggest to choose therapeutic options in relation to the dimension of the lesion, the stage of liver cirrhosis and the performance status, according to Barcelona Clinic Liver Cancer (BCLC) classification [8, 237, 238]. Current treatments for HCC include surgical resection, radio-frequency thermal ablation (RFTA), microwave ablation, alcoholization (percutaneous ethanol injection, PEI), liver transplantation, transarterial chemoembolization (TACE), transarterial radioembolization (TARE), targeted therapy with sorafenib and the recently approved first-line lenvatinib.

In elderly HCC patients, the therapeutic choices have not always been applied according to the suggestions of the guidelines as one of the main problems in the management of the treatment choice is the frequent presence of co-morbidities and usually a longer history of disease that limits the therapeutic options. Aging is in fact frequently associated with a functional reduction of the organs, including liver, conditions of disability, cognitive impairment, metabolic co-morbidities, such as diabetes or cardiovascular diseases, which contribute to define the elderly patients as “fragile”, whose prognosis is worse and with an expectation of survival that does not depend only on the staging of HCC. For these reasons, in the past patients in BCLC class 0 or A were treated with loco regional therapies such as RFTA, PEI or TACE instead of being treated with hepatectomy [239, 240]. Recently, the improvement of surgical procedures and perioperative management is, however, modifying this therapeutic approach.

## Surgical therapy

Most of the studies on liver surgery in populations of elderly or extremely old patients have been conducted in Japan, which is the country that has the highest life expectancy in the world 84.2 years [241].

Several studies have demonstrated that there are no substantial differences in complications among younger and elderly patients when the populations are correctly selected according to the guidelines [240–249], although some studies have reported a Grade 2 and 3 of complications according to the Clavien-Dindo classification [247, 248].

In the study by Wu et al., which reported on a series consisting mainly of octogenarians, there was a longer postoperative period than the young [249]. On the contrary, the study by Horiuchi et al. [250], assessing surgical outcome and long-term survival after elective hepatic resection for HCC in patients aged 80 years or more, found that incidence of postoperative complications, in-hospital mortality, and postoperative OS in the extremely elderly group were comparable with those of the elderly group. Finally, a recent Japanese nationwide study on the treatment optimization for HCC in the elderly, analyzing 6,940 HCC patients aged ≥75 years, concluded that hepatic resection was the best therapeutic option (vs RFTA and TACE) because decreased recurrence risk and improved OS in patients aged ≥75 years with primary HCC tumors <3.0 cm [251].

Anyways, globally considered, data on OS and duration of disease that these studies have reported as optimal, have some bias from a methodological point of view, therefore, it is reasonable to state that in elderly patients, selected according to the guidelines and carefully subjected to geriatric evaluation, surgery can be a valid therapeutic option [252].

## Radio-frequency thermal ablation

Radio-frequency thermal ablation (RFTA) is a very widespread method, which uses electric current and allows tumor necrosis due to heat production [253]. In elderly patients, especially if they have co-morbidities, RFTA is the therapy of choice in clinical practice. Several studies have shown survival rates comparable with those of the young [253–255], even if data on local progression are conflicting [254, 256]. Most of studies were conducted on Asian populations. However, a recently published paper conducted in Austria with a more innovative technique, confirmed previous observations obtained with RFTA, demonstrating that stereotactic radiofrequency ablation (SRFA) in octogenarians is a safe, feasible and useful option in the therapy of HCC with no significant difference in outcomes compared to a younger control group [257].

## Percutaneous ethanol injection

Percutaneous ethanol injection (PEI) therapy was the treatment of choice before the advent of RFTA and still today has some indications when for technical reasons RFTA is not feasible. In the elderly this approach was studied by Teratani et al. [258] who did not observe significantly different efficacy, safety profiles and survival rates in elderly HCC patients (aged ≥ 70 years) treated with PEI in comparison with younger patients.

## Transarterial chemoembolization

Transarterial chemoembolization (TACE) is nowadays considered the best therapeutic option for unresectable HCC in intermediate stage according to BCLC but in the past it was considered contraindicated in elderly patients [259]. Recent studies performed in western and eastern populations concluded that there were no observed differences in post-procedural complications depending on the age of patients, and that TACE was a safe and effective procedure in elderly patients [260, 261].

However, in a Japanese population of octogenarians, Cheng et al. [262] found that the performance status (ECOG 0) was the only independent prognostic significant factor, and that adverse events and OS rates were not different in the octogenarians.

## Targeted therapies

The frequent diagnosis of HCC at an advanced stage with the only therapeutic choice of systemic therapy in elderly has raised the question of how to manage this kind of therapy and its side effects in this category of patients. In fact, due to their more fragile conditions, they are generally considered more prone to suffer from toxic side effects which lead to dose reduction or interruption of therapy. Currently, treatment with sorafenib represents the oldest standard systemic therapy for advanced HCC in patients with preserved liver function (Child-Pugh class A). The trials of sorafenib, the SHARP and the Asia-Pacific trials, however, had median ages of patients of 64.9 and 51 years, respectively, even if in the subgroup analyses clinical benefits of the drug were proved similar in both younger (<65 years) and older (≥65 years) patients [263, 264]. Successively, the global investigation of therapeutic decision and of its treatment with sorafenib (GIDEON) study, a global, prospective, non-interventional study undertaken to evaluate the safety of sorafenib in patients with unresectable HCC in real-life practice, comparing safety profiles of sorafenib in patients <65 years and ≥65 years, showed that the incidence of side effects of any severity was independent of age [265]. In particular, in the Italian cohort of the GIDEON study sorafenib showed to be well tolerated and an effective treatment option in both younger and elderly patients, moreover elderly had longer OS than younger [266].

Although sorafenib was used as the only molecular targeted agent for HCC since 2007, other drugs were developed successively, but none of them passed from phase II or phase III clinical trials. Recently, novel drugs such as: regorafenib, cabozantinib, ramucirumab, nivolumab and pembrolizumab emerged from clinical trials for clinical use, but only as second line therapies. Indeed, lenvatinib is now feasible as an alternative to sorafenib as a first-line treatment for advanced HCC [267]. However, the safety and efficacy of lenvatinib in elderly patients with HCC has not been sufficiently investigated due to the limited numbers of elderly patients included in the trials. A recent Japanese study analyzed 100 patients with HCC who received lenvatinib, 50 elderly (age ≥ 75 years) and 50 non-elderly [268]. The authors found that there were no significant differences between the elderly and non-elderly groups in the frequency of adverse events and overall and progression-free survival, and they concluded that lenvatinib can be used safely and efficaciously regardless of age in patients with HCC [268].

## CONCLUSIONS

HCC represents a complicated disease as there is no single gene that is mutated in all HCC patients. The nature of evolution of HCC cells which can become invasive remains an area of intense research. HCC is a disease of aging as it is not frequently detected in younger people and may require the convergence of both complicated environmental and genetic mutational events. Treatment of elderly HCC patients (>75 years) remains a more difficult proposition as the safety and efficacy of the new target therapies have not been sufficiently investigated due to the limited numbers of elderly patients included in phase III clinical trials, therefore data from real-life practice are urgently needed.

Until recently, sorafenib was the only molecular targeted agent approved for treatment of advanced HCC patients. However, upon approval of lenvatinib in 2018 as first-line treatment, clinicians have another option of choice to treat patients with advanced HCC. In addition, with the approval of regorafenib, cabozantinib and ramucirumab as second-line treatments many patients, who progressed on sorafenib, nowadays may benefits for the increased treatment options. In near future, when ICIs, such as nivolumab and pembrolizumab, will eventually become available, more arms will be available to fight HCC. Consequently, a larger number of patients will benefit from these treatments, even using combination of them, i.e. ICIs and molecular targeted agents. In summary, the main issues, which remain, are the right drug, or combination of drugs, or sequential of drugs, for each given patient which may rely on personalized medicine based on genomic and other omics approaches.

## References

[1] Bray F, Ferlay J, Soerjomataram I, Siegel RL, Torre LA, Jemal A. Global cancer statistics 2018: GLOBOCAN estimates of incidence and mortality worldwide for 36 cancers in 185 countries. CA Cancer J Clin. 2018; 68:394–424. 10.3322/caac.21492 30207593

[2] Atyah M, Yin YR, Zhou CH, Zhou Q, Chen WY, Dong QZ, Ren N. Integrated analysis of the impact of age on genetic and clinical aspects of hepatocellular carcinoma. Aging (Albany NY). 2018; 10:2079–97. 10.18632/aging.101531 30125264 PMC6128442

[3] Wallace MC, Preen D, Jeffrey GP, Adams LA. The evolving epidemiology of hepatocellular carcinoma: a global perspective. Expert Rev Gastroenterol Hepatol. 2015; 9:765–79. 10.1586/17474124.2015.1028363 25827821

[4] El-Serag HB, Rudolph KL. Hepatocellular carcinoma: epidemiology and molecular carcinogenesis. Gastroenterology. 2007; 132:2557–76. 10.1053/j.gastro.2007.04.061 17570226

[5] El-Serag HB. Hepatocellular carcinoma. N Engl J Med. 2011; 365:1118–27. 10.1056/NEJMra1001683 21992124

[6] Bosetti C, Turati F, La Vecchia C. Hepatocellular carcinoma epidemiology. Best Pract Res Clin Gastroenterol. 2014; 28:753–70. 10.1016/j.bpg.2014.08.007 25260306

[7] Bucci L, Garuti F, Lenzi B, Pecorelli A, Farinati F, Giannini EG, Granito A, Ciccarese F, Rapaccini GL, Di Marco M, Caturelli E, Zoli M, Borzio F, et al, and Italian Liver Cancer (ITA.LI.CA) group. The evolutionary scenario of hepatocellular carcinoma in Italy: an update. Liver Int. 2017; 37:259–70. 10.1111/liv.13204 27427866

[8] Marrero JA, Kulik LM, Sirlin CB, Zhu AX, Finn RS, Abecassis MM, Roberts LR, Heimbach JK. Diagnosis, Staging, and Management of Hepatocellular Carcinoma: 2018 Practice Guidance by the American Association for the Study of Liver Diseases. Hepatology. 2018; 68:723–50. 10.1002/hep.29913 29624699

[9] El-Serag HB. Epidemiology of viral hepatitis and hepatocellular carcinoma. Gastroenterology. 2012; 142:1264–1273.e1. 10.1053/j.gastro.2011.12.061 22537432 PMC3338949

[10] Yang JD, Roberts LR. Hepatocellular carcinoma: A global view. Nat Rev Gastroenterol Hepatol. 2010; 7:448–58. 10.1038/nrgastro.2010.100 20628345 PMC3926946

[11] Soresi M, La Spada E, Giannitrapani L, Campagna E, Di Gesaro V, Granà W, Sandonato L, Brancatelli G, Rotolo G, Affronti A, Messina S, Montalto G. Hepatocellular carcinoma: comparison of two different periods at the same center. Eur J Intern Med. 2010; 21:127–30. 10.1016/j.ejim.2009.12.011 20206885

[12] Cazzagon N, Trevisani F, Maddalo G, Giacomin A, Vanin V, Pozzan C, Poggio PD, Rapaccini G, Nolfo AM, Benvegnù L, Zoli M, Borzio F, Giannini EG, et al, and Italian Liver Cancer (ITA.LI.CA) Group. Rise and fall of HCV-related hepatocellular carcinoma in Italy: a long-term survey from the ITA.LI.CA centres. Liver Int. 2013; 33:1420–27. 10.1111/liv.12208 23758775

[13] Mittal S, El-Serag HB. Epidemiology of hepatocellular carcinoma: consider the population. J Clin Gastroenterol. 2013 (47 Suppl); 47:S2–6. 10.1097/MCG.0b013e3182872f29 23632345 PMC3683119

[14] https://www.registri-tumori.it/cms/sites/default/files/pubblicazioni/2018_NumeriCancro-operatori.pdf.

[15] Stroffolini T, Sagnelli E, Gaeta GB, Sagnelli C, Andriulli A, Brancaccio G, Pirisi M, Colloredo G, Morisco F, Furlan C, Almasio PL, Almasio PL, Gaeta GB, et al, and EPACRON study group. Characteristics of liver cirrhosis in Italy: evidence for a decreasing role of HCV aetiology. Eur J Intern Med. 2017; 38:68–72. 10.1016/j.ejim.2016.10.012 27836249

[16] Bruno S, Stroffolini T, Colombo M, Bollani S, Benvegnù L, Mazzella G, Ascione A, Santantonio T, Piccinino F, Andreone P, Mangia A, Gaeta GB, Persico M, et al, and Italian Association of the Study of the Liver Disease (AISF). Sustained virological response to interferon-alpha is associated with improved outcome in HCV-related cirrhosis: a retrospective study. Hepatology. 2007; 45:579–87. 10.1002/hep.21492 17326216

[17] Younossi ZM, Otgonsuren M, Henry L, Venkatesan C, Mishra A, Erario M, Hunt S. Association of nonalcoholic fatty liver disease (NAFLD) with hepatocellular carcinoma (HCC) in the United States from 2004 to 2009. Hepatology. 2015; 62:1723–30. 10.1002/hep.28123 26274335

[18] Llovet JM, Montal R, Sia D, Finn RS. Molecular therapies and precision medicine for hepatocellular carcinoma. Nat Rev Clin Oncol. 2018; 15:599–616. 10.1038/s41571-018-0073-4 30061739 PMC12452113

[19] Li L, Wang H. Heterogeneity of liver cancer and personalized therapy. Cancer Lett. 2016; 379:191–97. 10.1016/j.canlet.2015.07.018 26213370

[20] Ally A, Balasundaram M, Carlsen R, Chuah E, Clarke A, Dhalla N, Holt RA, Jones SJ, Lee D, Ma Y, Marra MA, Mayo M, Moore RA, et al, and Cancer Genome Atlas Research Network. Electronic address: wheeler@bcm.edu, and Cancer Genome Atlas Research Network. Comprehensive and integrative genomic characterization of hepatocellular carcinoma. Cell. 2017; 169:1327–1341.e23. 10.1016/j.cell.2017.05.046 28622513 PMC5680778

[21] Hoshida Y, Nijman SM, Kobayashi M, Chan JA, Brunet JP, Chiang DY, Villanueva A, Newell P, Ikeda K, Hashimoto M, Watanabe G, Gabriel S, Friedman SL, et al. Integrative transcriptome analysis reveals common molecular subclasses of human hepatocellular carcinoma. Cancer Res. 2009; 69:7385–92. 10.1158/0008-5472.CAN-09-1089 19723656 PMC3549578

[22] Cervello M, McCubrey JA, Cusimano A, Lampiasi N, Azzolina A, Montalto G. Targeted therapy for hepatocellular carcinoma: novel agents on the horizon. Oncotarget. 2012; 3:236–60. 10.18632/oncotarget.466 22470194 PMC3359882

[23] Cervello M, Emma MR, Augello G, Balasus D, Azzolina A, McCubrey JA, Cusimano A. From targets to targeted therapies in hepatocellular carcinoma. For Immunopathol Dis Therap. 2014; 5:145–94. 10.1615/ForumImmunDisTher.2015013982

[24] Steelman LS, Fitzgerald T, Lertpiriyapong K, Cocco L, Follo MY, Martelli AM, Neri LM, Marmiroli S, Libra M, Candido S, Nicoletti F, Scalisi A, Fenga C, et al. Critical roles of EGFR family members in breast cancer and breast cancer stem cells: targets for therapy. Curr Pharm Des. 2016; 22:2358–88. 10.2174/1381612822666160304151011 26947958

[25] Davis NM, Sokolosky M, Stadelman K, Abrams SL, Libra M, Candido S, Nicoletti F, Polesel J, Maestro R, D’Assoro A, Drobot L, Rakus D, Gizak A, et al. Deregulation of the EGFR/PI3K/PTEN/Akt/mTORC1 pathway in breast cancer: possibilities for therapeutic intervention. Oncotarget. 2014; 5:4603–50. 10.18632/oncotarget.2209 25051360 PMC4148087

[26] Fuchs BC, Hoshida Y, Fujii T, Wei L, Yamada S, Lauwers GY, McGinn CM, DePeralta DK, Chen X, Kuroda T, Lanuti M, Schmitt AD, Gupta S, et al. Epidermal growth factor receptor inhibition attenuates liver fibrosis and development of hepatocellular carcinoma. Hepatology. 2014; 59:1577–90. 10.1002/hep.26898 24677197 PMC4086837

[27] Komposch K, Sibilia M. EGFR signaling in liver diseases. Int J Mol Sci. 2015; 17:E30. 10.3390/ijms17010030 26729094 PMC4730276

[28] Song P, Yang J, Li X, Huang H, Guo X, Zhou G, Xu X, Cai Y, Zhu M, Wang P, Zhao S, Zhang D. Hepatocellular carcinoma treated with anti-epidermal growth factor receptor antibody nimotuzumab: A case report. Medicine (Baltimore). 2017; 96:e8122. 10.1097/MD.0000000000008122 28953642 PMC5626285

[29] O’Dwyer PJ, Giantonio BJ, Levy DE, Kauh JS, Fitzgerald DB, Benson III AB. Gefitinib in advanced unresectable hepatocellular carcinoma: Results from the eastern cooperative oncology group’s study E1203. J Clin Oncol. 2006; 24:4143–4143. https://ascopubs.org/doi/abs/10.1200/jco.2006.24.18_suppl.414316896003

[30] Chiorean EG, Ramasubbaiah R, Yu M, Picus J, Bufill JA, Tong Y, Coleman N, Johnston EL, Currie C, Loehrer PJ. Phase II trial of erlotinib and docetaxel in advanced and refractory hepatocellular and biliary cancers: Hoosier Oncology Group GI06-101. Oncologist. 2012; 17:13–e23. 10.1634/theoncologist.2011-0253 22210086 PMC3267812

[31] Philip PA, Mahoney MR, Holen KD, Northfelt DW, Pitot HC, Picus J, Flynn PJ, Erlichman C. Phase 2 study of bevacizumab plus erlotinib in patients with advanced hepatocellular cancer. Cancer. 2012; 118:2424–30. 10.1002/cncr.26556 21953248 PMC3896238

[32] Blume-Jensen P, Hunter T. Oncogenic kinase signalling. Nature. 2001; 411:355–65. 10.1038/35077225 11357143

[33] Jansson S, Aaltonen K, Bendahl PO, Falck AK, Karlsson M, Pietras K, Rydén L. The PDGF pathway in breast cancer is linked to tumour aggressiveness, triple-negative subtype and early recurrence. Breast Cancer Res Treat. 2018; 169:231–41. 10.1007/s10549-018-4664-7 29380207 PMC5945746

[34] Wang JX, Zhou JF, Huang FK, Zhang L, He QL, Qian HY, Lai HL. GLI2 induces PDGFRB expression and modulates cancer stem cell properties of gastric cancer. Eur Rev Med Pharmacol Sci. 2017; 21:3857–65. 28975979

[35] Fukahi K, Fukasawa M, Neufeld G, Itakura J, Korc M. Aberrant expression of neuropilin-1 and -2 in human pancreatic cancer cells. Clin Cancer Res. 2004; 10:581–90. 10.1158/1078-0432.CCR-0930-03 14760080

[36] Shida A, Fujioka S, Kobayashi K, Ishibashi Y, Nimura H, Mitsumori N, Yanaga K. Expression of vascular endothelial growth factor (VEGF)-C and -D in gastric carcinoma. Int J Clin Oncol. 2006; 11:38–43. 10.1007/s10147-005-0528-3 16508727

[37] Martins SF, Garcia EA, Luz MA, Pardal F, Rodrigues M, Filho AL. Clinicopathological correlation and prognostic significance of VEGF-A, VEGF-C, VEGFR-2 and VEGFR-3 expression in colorectal cancer. Cancer Genomics Proteomics. 2013; 10:55–67. 23603341

[38] Cao G, Li X, Qin C, Li J. Prognostic Value of VEGF in hepatocellular carcinoma patients treated with sorafenib: A meta-analysis. Med Sci Monit. 2015; 21:3144–51. 10.12659/MSM.894617 26476711 PMC4617189

[39] Chen B, Liu J, Wang X, Shen Q, Li C, Dai C. Co-expression of PDGF-B and VEGFR-3 strongly correlates with poor prognosis in hepatocellular carcinoma patients after hepatectomy. Clin Res Hepatol Gastroenterol. 2018; 42:126–33. 10.1016/j.clinre.2016.11.006 29273278

[40] Chu JS, Ge FJ, Zhang B, Wang Y, Silvestris N, Liu LJ, Zhao CH, Lin L, Brunetti AE, Fu YL, Wang J, Paradiso A, Xu JM. Expression and prognostic value of VEGFR-2, PDGFR-β, and c-Met in advanced hepatocellular carcinoma. J Exp Clin Cancer Res. 2013; 32:16. 10.1186/1756-9966-32-16 23552472 PMC3623756

[41] Yu JH, Kim JM, Kim JK, Choi SJ, Lee KS, Lee JW, Chang HY, Lee JI. Platelet-derived growth factor receptor α in hepatocellular carcinoma is a prognostic marker independent of underlying liver cirrhosis. Oncotarget. 2017; 8:39534–46. 10.18632/oncotarget.17134 28465473 PMC5503630

[42] Aryal B, Yamakuchi M, Shimizu T, Kadono J, Furoi A, Gejima K, Komokata T, Koriyama C, Hashiguchi T, Imoto Y. Predictive value of diminished serum PDGF-BB after curative resection of hepatocellular cancer. J Oncol. 2019; 2019:1925315. 10.1155/2019/1925315 30723501 PMC6339767

[43] Govaere O, Petz M, Wouters J, Vandewynckel YP, Scott EJ, Topal B, Nevens F, Verslype C, Anstee QM, Van Vlierberghe H, Mikulits W, Roskams T. The PDGFRα-laminin B1-keratin 19 cascade drives tumor progression at the invasive front of human hepatocellular carcinoma. Oncogene. 2017; 36:6605–16. 10.1038/onc.2017.260 28783171 PMC5702717

[44] Rimassa L, Santoro A. Sorafenib therapy in advanced hepatocellular carcinoma: the SHARP trial. Expert Rev Anticancer Ther. 2009; 9:739–45. 10.1586/era.09.41 19496710

[45] Finn RS, Merle P, Granito A, Huang YH, Bodoky G, Pracht M, Yokosuka O, Rosmorduc O, Gerolami R, Caparello C, Cabrera R, Chang C, Sun W, et al. Outcomes of sequential treatment with sorafenib followed by regorafenib for HCC: additional analyses from the phase III RESORCE trial. J Hepatol. 2018; 69:353–58. 10.1016/j.jhep.2018.04.010 29704513

[46] Teufel M, Köchert K, Meinhardt G, Bruix J. Efficacy of regorafenib (reg) in patients with hepatocellular carcinoma (hcc) in the phase III resorce trial according to alpha-fetoprotein (afp) and c-met levels as predictors of poor prognosis. J Clin Oncol. 2017 (15_Suppl); 35:4078 10.1200/JCO.2017.35.15_suppl.407829064744

[47] Potapova O, Laird AD, Nannini MA, Barone A, Li G, Moss KG, Cherrington JM, Mendel DB. Contribution of individual targets to the antitumor efficacy of the multitargeted receptor tyrosine kinase inhibitor SU11248. Mol Cancer Ther. 2006; 5:1280–89. 10.1158/1535-7163.MCT-03-0156 16731761

[48] Zhu AX, Sahani DV, Duda DG, di Tomaso E, Ancukiewicz M, Catalano OA, Sindhwani V, Blaszkowsky LS, Yoon SS, Lahdenranta J, Bhargava P, Meyerhardt J, Clark JW, et al. Efficacy, safety, and potential biomarkers of sunitinib monotherapy in advanced hepatocellular carcinoma: a phase II study. J Clin Oncol. 2009; 27:3027–35. 10.1200/JCO.2008.20.9908 19470923 PMC2702235

[49] Faivre S, Raymond E, Boucher E, Douillard J, Lim HY, Kim JS, Zappa M, Lanzalone S, Lin X, Deprimo S, Harmon C, Ruiz-Garcia A, Lechuga MJ, Cheng AL. Safety and efficacy of sunitinib in patients with advanced hepatocellular carcinoma: an open-label, multicentre, phase II study. Lancet Oncol. 2009; 10:794–800. 10.1016/S1470-2045(09)70171-8 19586800

[50] Koeberle D, Montemurro M, Samaras P, Majno P, Simcock M, Limacher A, Lerch S, Kovàcs K, Inauen R, Hess V, Saletti P, Borner M, Roth A, Bodoky G. Continuous Sunitinib treatment in patients with advanced hepatocellular carcinoma: a Swiss Group for Clinical Cancer Research (SAKK) and Swiss Association for the Study of the Liver (SASL) multicenter phase II trial (SAKK 77/06). Oncologist. 2010; 15:285–92. 10.1634/theoncologist.2009-0316 20203173 PMC3227954

[51] Toh HC, Chen PJ, Carr BI, Knox JJ, Gill S, Ansell P, McKeegan EM, Dowell B, Pedersen M, Qin Q, Qian J, Scappaticci FA, Ricker JL, et al. Phase 2 trial of linifanib (ABT-869) in patients with unresectable or metastatic hepatocellular carcinoma. Cancer. 2013; 119:380–87. 10.1002/cncr.27758 22833179

[52] Cainap C, Qin S, Huang WT, Chung IJ, Pan H, Cheng Y, Kudo M, Kang YK, Chen PJ, Toh HC, Gorbunova V, Eskens FA, Qian J, et al. Linifanib versus Sorafenib in patients with advanced hepatocellular carcinoma: results of a randomized phase III trial. J Clin Oncol. 2015; 33:172–79. 10.1200/JCO.2013.54.3298 25488963 PMC4279237

[53] Park JW, Finn RS, Kim JS, Karwal M, Li RK, Ismail F, Thomas M, Harris R, Baudelet C, Walters I, Raoul JL. Phase II, open-label study of brivanib as first-line therapy in patients with advanced hepatocellular carcinoma. Clin Cancer Res. 2011; 17:1973–83. 10.1158/1078-0432.CCR-10-2011 21349999

[54] Finn RS, Kang YK, Mulcahy M, Polite BN, Lim HY, Walters I, Baudelet C, Manekas D, Park JW. Phase II, open-label study of brivanib as second-line therapy in patients with advanced hepatocellular carcinoma. Clin Cancer Res. 2012; 18:2090–98. 10.1158/1078-0432.CCR-11-1991 22238246

[55] Johnson PJ, Qin S, Park JW, Poon RT, Raoul JL, Philip PA, Hsu CH, Hu TH, Heo J, Xu J, Lu L, Chao Y, Boucher E, et al. Brivanib versus sorafenib as first-line therapy in patients with unresectable, advanced hepatocellular carcinoma: results from the randomized phase III BRISK-FL study. J Clin Oncol. 2013; 31:3517–24. 10.1200/JCO.2012.48.4410 23980084

[56] Llovet JM, Decaens T, Raoul JL, Boucher E, Kudo M, Chang C, Kang YK, Assenat E, Lim HY, Boige V, Mathurin P, Fartoux L, Lin DY, et al. Brivanib in patients with advanced hepatocellular carcinoma who were intolerant to sorafenib or for whom sorafenib failed: results from the randomized phase III BRISK-PS study. J Clin Oncol. 2013; 31:3509–16. 10.1200/JCO.2012.47.3009 23980090

[57] Kudo M, Han G, Finn RS, Poon RT, Blanc JF, Yan L, Yang J, Lu L, Tak WY, Yu X, Lee JH, Lin SM, Wu C, et al. Brivanib as adjuvant therapy to transarterial chemoembolization in patients with hepatocellular carcinoma: A randomized phase III trial. Hepatology. 2014; 60:1697–707. 10.1002/hep.27290 24996197

[58] Xiang Q, Chen W, Ren M, Wang J, Zhang H, Deng DY, Zhang L, Shang C, Chen Y. Cabozantinib suppresses tumor growth and metastasis in hepatocellular carcinoma by a dual blockade of VEGFR2 and MET. Clin Cancer Res. 2014; 20:2959–70. 10.1158/1078-0432.CCR-13-2620 24700742

[59] Abou-Alfa GK, Meyer T, Cheng AL, El-Khoueiry AB, Rimassa L, Ryoo BY, Cicin I, Merle P, Chen Y, Park JW, Blanc JF, Bolondi L, Klümpen HJ, et al. Cabozantinib in patients with advanced and progressing hepatocellular carcinoma. N Engl J Med. 2018; 379:54–63. 10.1056/NEJMoa1717002 29972759 PMC7523244

[60] Kudo M, Finn RS, Qin S, Han KH, Ikeda K, Piscaglia F, Baron A, Park JW, Han G, Jassem J, Blanc JF, Vogel A, Komov D, et al. Lenvatinib versus sorafenib in first-line treatment of patients with unresectable hepatocellular carcinoma: a randomised phase 3 non-inferiority trial. Lancet. 2018; 391:1163–73. 10.1016/S0140-6736(18)30207-1 29433850

[61] Finn RS, Bentley G, Britten CD, Amado R, Busuttil RW. Targeting vascular endothelial growth factor with the monoclonal antibody bevacizumab inhibits human hepatocellular carcinoma cells growing in an orthotopic mouse model. Liver Int. 2009; 29:284–90. 10.1111/j.1478-3231.2008.01762.x 18482274

[62] Xiong YQ, Sun HC, Zhu XD, Zhang W, Zhuang PY, Zhang JB, Xu HX, Kong LQ, Wu WZ, Qin LX, Tang ZY. Bevacizumab enhances chemosensitivity of hepatocellular carcinoma to adriamycin related to inhibition of survivin expression. J Cancer Res Clin Oncol. 2011; 137:505–12. 10.1007/s00432-010-0914-8 20490863 PMC11828078

[63] Boige V, Malka D, Bourredjem A, Dromain C, Baey C, Jacques N, Pignon JP, Vimond N, Bouvet-Forteau N, De Baere T, Ducreux M, Farace F. Efficacy, safety, and biomarkers of single-agent bevacizumab therapy in patients with advanced hepatocellular carcinoma. Oncologist. 2012; 17:1063–72. 10.1634/theoncologist.2011-0465 22707516 PMC3425524

[64] Hubbard JM, Mahoney MR, Loui WS, Roberts LR, Smyrk TC, Gatalica Z, Borad M, Kumar S, Alberts SR. Phase I/II randomized trial of sorafenib and bevacizumab as first-line therapy in patients with locally advanced or metastatic hepatocellular carcinoma: north central cancer treatment group trial N0745 (Alliance). Target Oncol. 2017; 12:201–09. 10.1007/s11523-016-0467-0 27943153 PMC5586602

[65] Hsu CH, Yang TS, Hsu C, Toh HC, Epstein RJ, Hsiao LT, Chen PJ, Lin ZZ, Chao TY, Cheng AL. Efficacy and tolerability of bevacizumab plus capecitabine as first-line therapy in patients with advanced hepatocellular carcinoma. Br J Cancer. 2010; 102:981–86. 10.1038/sj.bjc.6605580 20160718 PMC2844032

[66] Thomas MB, Morris JS, Chadha R, Iwasaki M, Kaur H, Lin E, Kaseb A, Glover K, Davila M, Abbruzzese J. Phase II trial of the combination of bevacizumab and erlotinib in patients who have advanced hepatocellular carcinoma. J Clin Oncol. 2009; 27:843–50. 10.1200/JCO.2008.18.3301 19139433

[67] Pinter M, Ulbrich G, Sieghart W, Kölblinger C, Reiberger T, Li S, Ferlitsch A, Müller C, Lammer J, Peck-Radosavljevic M. Hepatocellular carcinoma: A phase II randomized controlled double-blind trial of transarterial chemoembolization in combination with biweekly intravenous administration of bevacizumab or a placebo. Radiology. 2015; 277:903–12. 10.1148/radiol.2015142140 26131911

[68] Zhu AX, Park JO, Ryoo BY, Yen CJ, Poon R, Pastorelli D, Blanc JF, Chung HC, Baron AD, Pfiffer TE, Okusaka T, Kubackova K, Trojan J, et al, and REACH Trial Investigators. Ramucirumab versus placebo as second-line treatment in patients with advanced hepatocellular carcinoma following first-line therapy with sorafenib (REACH): a randomised, double-blind, multicentre, phase 3 trial. Lancet Oncol. 2015; 16:859–70. 10.1016/S1470-2045(15)00050-9 26095784

[69] Zhu AX, Kang YK, Yen CJ, Finn RS, Galle PR, Llovet JM, Assenat E, Brandi G, Pracht M, Lim HY, Rau KM, Motomura K, Ohno I, et al, and REACH-2 study investigators. Ramucirumab after sorafenib in patients with advanced hepatocellular carcinoma and increased α-fetoprotein concentrations (REACH-2): a randomised, double-blind, placebo-controlled, phase 3 trial. Lancet Oncol. 2019; 20:282–96. 10.1016/S1470-2045(18)30937-9 30665869

[70] Lin BC, Wang M, Blackmore C, Desnoyers LR. Liver-specific activities of FGF19 require Klotho beta. J Biol Chem. 2007; 282:27277–84. 10.1074/jbc.M704244200 17627937

[71] Tiong KH, Tan BS, Choo HL, Chung FF, Hii LW, Tan SH, Khor NT, Wong SF, See SJ, Tan YF, Rosli R, Cheong SK, Leong CO. Fibroblast growth factor receptor 4 (FGFR4) and fibroblast growth factor 19 (FGF19) autocrine enhance breast cancer cells survival. Oncotarget. 2016; 7:57633–50. 10.18632/oncotarget.9328 27192118 PMC5295378

[72] Wesche J, Haglund K, Haugsten EM. Fibroblast growth factors and their receptors in cancer. Biochem J. 2011; 437:199–213. 10.1042/BJ20101603 21711248

[73] Wu X, Ge H, Lemon B, Vonderfecht S, Weiszmann J, Hecht R, Gupte J, Hager T, Wang Z, Lindberg R, Li Y. FGF19-induced hepatocyte proliferation is mediated through FGFR4 activation. J Biol Chem. 2010; 285:5165–70. 10.1074/jbc.M109.068783 20018895 PMC2820743

[74] Gao L, Wang X, Tang Y, Huang S, Hu CA, Teng Y. FGF19/FGFR4 signaling contributes to the resistance of hepatocellular carcinoma to sorafenib. J Exp Clin Cancer Res. 2017; 36:8. 10.1186/s13046-016-0478-9 28069043 PMC5223586

[75] Miura S, Mitsuhashi N, Shimizu H, Kimura F, Yoshidome H, Otsuka M, Kato A, Shida T, Okamura D, Miyazaki M. Fibroblast growth factor 19 expression correlates with tumor progression and poorer prognosis of hepatocellular carcinoma. BMC Cancer. 2012; 12:56. 10.1186/1471-2407-12-56 22309595 PMC3293719

[76] Lin ZZ, Hsu C, Jeng YM, Hu FC, Pan HW, Wu YM, Hsu HC, Cheng AL. Klotho-beta and fibroblast growth factor 19 expression correlates with early recurrence of resectable hepatocellular carcinoma. Liver Int. 2019; 39:1682–91. 10.1111/liv.14055 30698907

[77] Perera TP, Jovcheva E, Mevellec L, Vialard J, De Lange D, Verhulst T, Paulussen C, Van De Ven K, King P, Freyne E, Rees DC, Squires M, Saxty G, et al. Discovery and pharmacological characterization of JNJ-42756493 (Erdafitinib), a functionally selective small-molecule FGFR family inhibitor. Mol Cancer Ther. 2017; 16:1010–20. 10.1158/1535-7163.MCT-16-0589 28341788

[78] Zhao G, Li WY, Chen D, Henry JR, Li HY, Chen Z, Zia-Ebrahimi M, Bloem L, Zhai Y, Huss K, Peng SB, McCann DJ. A novel, selective inhibitor of fibroblast growth factor receptors that shows a potent broad spectrum of antitumor activity in several tumor xenograft models. Mol Cancer Ther. 2011; 10:2200–10. 10.1158/1535-7163.MCT-11-0306 21900693

[79] Hagel M, Miduturu C, Sheets M, Rubin N, Weng W, Stransky N, Bifulco N, Kim JL, Hodous B, Brooijmans N, Shutes A, Winter C, Lengauer C, et al. First selective small molecule inhibitor of FGFR4 for the treatment of hepatocellular carcinomas with an activated FGFR4 signaling pathway. Cancer Discov. 2015; 5:424–37. 10.1158/2159-8290.CD-14-1029 25776529

[80] Kim RD, Sarker D, Meyer T, Yau T, Macarulla T, Park JW, Choo SP, Hollebecque A, Sung MW, Lim HY, Mazzaferro V, Trojan J, Zhu AX, et al. First-in-Human Phase I Study of Fisogatinib (BLU-554) Validates Aberrant FGF19 Signaling as a Driver Event in Hepatocellular Carcinoma. Cancer Discov. 2019; 9:1696–707. 10.1158/2159-8290.CD-19-0555 31575541

[81] Joshi JJ, Coffey H, Corcoran E, Tsai J, Huang CL, Ichikawa K, Prajapati S, Hao MH, Bailey S, Wu J, Rimkunas V, Karr C, Subramanian V, et al. H3B-6527 Is a potent and selective inhibitor of FGFR4 in FGF19-driven hepatocellular carcinoma. Cancer Res. 2017; 77:6999–7013. 10.1158/0008-5472.CAN-17-1865 29247039

[82] McCubrey JA, Steelman LS, Chappell WH, Abrams SL, Montalto G, Cervello M, Nicoletti F, Fagone P, Malaponte G, Mazzarino MC, Candido S, Libra M, Bäsecke J, et al. Mutations and deregulation of Ras/Raf/MEK/ERK and PI3K/PTEN/Akt/mTOR cascades which alter therapy response. Oncotarget. 2012; 3:954–87. 10.18632/oncotarget.652 23006971 PMC3660063

[83] Steelman LS, Chappell WH, Abrams SL, Kempf RC, Long J, Laidler P, Mijatovic S, Maksimovic-Ivanic D, Stivala F, Mazzarino MC, Donia M, Fagone P, Malaponte G, et al. Roles of the Raf/MEK/ERK and PI3K/PTEN/Akt/mTOR pathways in controlling growth and sensitivity to therapy-implications for cancer and aging. Aging (Albany NY). 2011; 3:192–222. 10.18632/aging.100296 21422497 PMC3091517

[84] McCubrey JA, Steelman LS, Chappell WH, Abrams SL, Franklin RA, Montalto G, Cervello M, Libra M, Candido S, Malaponte G, Mazzarino MC, Fagone P, Nicoletti F, et al. Ras/Raf/MEK/ERK and PI3K/PTEN/Akt/mTOR cascade inhibitors: how mutations can result in therapy resistance and how to overcome resistance. Oncotarget. 2012; 3:1068–111. 10.18632/oncotarget.659 23085539 PMC3717945

[85] McCubrey JA, Steelman LS, Kempf CR, Chappell WH, Abrams SL, Stivala F, Malaponte G, Nicoletti F, Libra M, Bäsecke J, Maksimovic-Ivanic D, Mijatovic S, Montalto G, et al. Therapeutic resistance resulting from mutations in Raf/MEK/ERK and PI3K/PTEN/Akt/mTOR signaling pathways. J Cell Physiol. 2011; 226:2762–81. 10.1002/jcp.22647 21302297

[86] Lee JT, McCubrey JA. BAY-43-9006 Bayer/Onyx. Curr Opin Investig Drugs. 2003; 4:757–63. 12901237

[87] Bi F, Qiu M, Chai X, Niu J, Ding Y, Bai Y, Wu L, Shentu J, Hao P, Chen J, Li Q. A multicenter phase II study of donafenib in patients with advanced hepatocellular carcinoma. J Clin Oncol. 2017 (15_Suppl); 35:e1568 10.1200/JCO.2017.35.15_suppl.e15682

[88] Hsu CY, Shen YC, Yu CW, Hsu C, Hu FC, Hsu CH, Chen BB, Wei SY, Cheng AL, Shih TT. Dynamic contrast-enhanced magnetic resonance imaging biomarkers predict survival and response in hepatocellular carcinoma patients treated with sorafenib and metronomic tegafur/uracil. J Hepatol. 2011; 55:858–65. 10.1016/j.jhep.2011.01.032 21338641

[89] Tai WM, Yong WP, Lim C, Low LS, Tham CK, Koh TS, Ng QS, Wang WW, Wang LZ, Hartano S, Thng CH, Huynh H, Lim KT, et al. A phase Ib study of selumetinib (AZD6244, ARRY-142886) in combination with sorafenib in advanced hepatocellular carcinoma (HCC). Ann Oncol. 2016; 27:2210–15. 10.1093/annonc/mdw415 27681866

[90] Adjei AA, Richards DA, El-Khoueiry A, Braiteh F, Becerra CH, Stephenson JJ Jr, Hezel AF, Sherman M, Garbo L, Leffingwell DP, Iverson C, Miner JN, Shen Z, et al. A phase I study of the safety, pharmacokinetic, and pharmodyamics of combination therapy with rfametinb plus sorafenib in patients with advanced cancer. Clin Cancer Res. 2016; 22:2368–76. 10.1158/1078-0432.CCR-15-1681 26644411 PMC7519584

[91] Lim HY, Heo J, Choi HJ, Lin CY, Yoon JH, Hsu C, Rau KM, Poon RT, Yeo W, Park JW, Tay MH, Hsieh WS, Kappeler C, et al. A phase II study of the efficacy and safety of the combination therapy of the MEK inhibitor refametinib (BAY 86-9766) plus sorafenib for Asian patients with unresectable hepatocellular carcinoma. Clin Cancer Res. 2014; 20:5976–85. 10.1158/1078-0432.CCR-13-3445 25294897

[92] Milella M, Falcone I, Conciatori F, Matteoni S, Sacconi A, De Luca T, Bazzichetto C, Corbo V, Simbolo M, Sperduti I, Benfante A, Del Curatolo A, Cesta Incani U, et al. PTEN status is a crucial determinant of the functional outcome of combined MEK and mTOR inhibition in cancer. Sci Rep. 2017; 7:43013. 10.1038/srep43013 28220839 PMC5318947

[93] Augello G, Puleio R, Emma MR, Cusimano A, Loria GR, McCubrey JA, Montalto G, Cervello M. A PTEN inhibitor displays preclinical activity against hepatocarcinoma cells. Cell Cycle. 2016; 15:573–83. 10.1080/15384101.2016.1138183 26794644 PMC5056616

[94] Simioni C, Cani A, Martelli AM, Zauli G, Alameen AA, Ultimo S, Tabellini G, McCubrey JA, Capitani S, Neri LM. The novel dual PI3K/mTOR inhibitor NVP-BGT226 displays cytotoxic activity in both normoxic and hypoxic hepatocarcinoma cells. Oncotarget. 2015; 6:17147–60. 10.18632/oncotarget.3940 26003166 PMC4627298

[95] Chiarini F, Evangelisti C, McCubrey JA, Martelli AM. Current treatment strategies for inhibiting mTOR in cancer. Trends Pharmacol Sci. 2015; 36:124–35. 10.1016/j.tips.2014.11.004 25497227

[96] Shiah HS, Chen CY, Dai CY, Hsiao CF, Lin YJ, Su WC, Chang JY, Whang-Peng J, Lin PW, Huang JD, Chen LT. Randomised clinical trial: comparison of two everolimus dosing schedules in patients with advanced hepatocellular carcinoma. Aliment Pharmacol Ther. 2013; 37:62–73. 10.1111/apt.12132 23134470

[97] Zhu AX, Kudo M, Assenat E, Cattan S, Kang YK, Lim HY, Poon RT, Blanc JF, Vogel A, Chen CL, Dorval E, Peck-Radosavljevic M, Santoro A, et al. Effect of everolimus on survival in advanced hepatocellular carcinoma after failure of sorafenib: the EVOLVE-1 randomized clinical trial. JAMA. 2014; 312:57–67. 10.1001/jama.2014.7189 25058218

[98] Koeberle D, Dufour JF, Demeter G, Li Q, Ribi K, Samaras P, Saletti P, Roth AD, Horber D, Buehlmann M, Wagner AD, Montemurro M, Lakatos G, et al, and Swiss Group for Clinical Cancer Research (SAKK). Sorafenib with or without everolimus in patients with advanced hepatocellular carcinoma (HCC): a randomized multicenter, multinational phase II trial (SAKK 77/08 and SASL 29). Ann Oncol. 2016; 27:856–61. 10.1093/annonc/mdw054 26884590

[99] Knox JJ, Qin R, Strosberg JR, Tan B, Kaubisch A, El-Khoueiry AB, Bekaii-Saab TS, Rousey SR, Chen HX, Erlichman C. A phase II trial of bevacizumab plus temsirolimus in patients with advanced hepatocellular carcinoma. Invest New Drugs. 2015; 33:241–46. 10.1007/s10637-014-0169-3 25318437

[100] Choo SP, Chowbay B, Ng QS, Thng CH, Lim C, Hartono S, Koh TS, Huynh H, Poon D, Ang MK, Chang S, Toh HC. A Phase 1 dose-finding and pharmacodynamic study of rapamycin in combination with bevacizumab in patients with unresectable hepatocellular carcinoma. Eur J Cancer. 2013; 49:999–1008. 10.1016/j.ejca.2012.11.008 23265712

[101] Geissler EK, Schnitzbauer AA, Zülke C, Lamby PE, Proneth A, Duvoux C, Burra P, Jauch KW, Rentsch M, Ganten TM, Schmidt J, Settmacher U, Heise M, et al. Sirolimus use in liver transplant recipients with hepatocellular carcinoma: A Randomized, multicenter, open-label phase 3 trial. Transplantation. 2016; 100:116–25. 10.1097/TP.0000000000000965 26555945 PMC4683033

[102] Moustakas A, Heldin CH. The regulation of TGFbeta signal transduction. Development. 2009; 136:3699–714. 10.1242/dev.030338 19855013

[103] Pickup M, Novitskiy S, Moses HL. The roles of TGFβ in the tumour microenvironment. Nat Rev Cancer. 2013; 13:788–99. 10.1038/nrc3603 24132110 PMC4025940

[104] Morikawa M, Derynck R, Miyazono K. TGF-β and the TGF-β family: context-dependent roles in cell and tissue physiology. Cold Spring Harb Perspect Biol. 2016; 8:a021873. 10.1101/cshperspect.a021873 27141051 PMC4852809

[105] Hinck AP, Mueller TD, Springer TA. Structural biology and evolution of the TGF-β family. Cold Spring Harb Perspect Biol. 2016; 8:a022103. 10.1101/cshperspect.a022103 27638177 PMC5131774

[106] Principe DR, Doll JA, Bauer J, Jung B, Munshi HG, Bartholin L, Pasche B, Lee C, Grippo PJ. TGF-β: duality of function between tumor prevention and carcinogenesis. J Natl Cancer Inst. 2014; 106:djt369. 10.1093/jnci/djt369 24511106 PMC3952197

[107] Jakowlew SB. Transforming growth factor-beta in cancer and metastasis. Cancer Metastasis Rev. 2006; 25:435–57. 10.1007/s10555-006-9006-2 16951986

[108] Tian M, Neil JR, Schiemann WP. Transforming growth factor-β and the hallmarks of cancer. Cell Signal. 2011; 23:951–62. 10.1016/j.cellsig.2010.10.015 20940046 PMC3076078

[109] Drabsch Y, ten Dijke P. TGF-β signalling and its role in cancer progression and metastasis. Cancer Metastasis Rev. 2012; 31:553–68. 10.1007/s10555-012-9375-7 22714591

[110] Wendt MK, Tian M, Schiemann WP. Deconstructing the mechanisms and consequences of TGF-β-induced EMT during cancer progression. Cell Tissue Res. 2012; 347:85–101. 10.1007/s00441-011-1199-1 21691718 PMC3723118

[111] Nakao A, Imamura T, Souchelnytskyi S, Kawabata M, Ishisaki A, Oeda E, Tamaki K, Hanai J, Heldin CH, Miyazono K, ten Dijke P. TGF-β receptor-mediated signalling through Smad2, Smad3 and Smad4. EMBO J. 1997; 16:5353–62. 10.1093/emboj/16.17.5353 9311995 PMC1170167

[112] Budi EH, Duan D, Derynck R. Transforming growth factor-β receptors and Smads: regulatory complexity and functional versatility. Trends Cell Biol. 2017; 27:658–72. 10.1016/j.tcb.2017.04.005 28552280

[113] Derynck R, Zhang YE. Smad-dependent and Smad-independent pathways in TGF-β family signalling. Nature. 2003; 425:577–84. 10.1038/nature02006 14534577

[114] Heldin CH, Landström M, Moustakas A. Mechanism of TGF-β signaling to growth arrest, apoptosis, and epithelial-mesenchymal transition. Curr Opin Cell Biol. 2009; 21:166–76. 10.1016/j.ceb.2009.01.021 19237272

[115] Zhang YE. Non-Smad signaling pathways of the TGF- β family. Cold Spring Harb Perspect Biol. 2017; 9:a022129. 10.1101/cshperspect.a022129 27864313 PMC5287080

[116] Lee D, Chung YH, Kim JA, Lee YS, Lee D, Jang MK, Kim KM, Lim YS, Lee HC, Lee YS. Transforming growth factor beta 1 overexpression is closely related to invasiveness of hepatocellular carcinoma. Oncology. 2012; 82:11–18. 10.1159/000335605 22269311

[117] Okumoto K, Hattori E, Tamura K, Kiso S, Watanabe H, Saito K, Saito T, Togashi H, Kawata S. Possible contribution of circulating transforming growth factor-beta1 to immunity and prognosis in unresectable hepatocellular carcinoma. Liver Int. 2004; 24:21–28. 10.1111/j.1478-3231.2004.00882.x 15101997

[118] Dituri F, Serio G, Filannino D, Mascolo A, Sacco R, Villa E, Giannelli G. Circulating TGF-β1-related biomarkers in patients with hepatocellular carcinoma and their association with HCC staging scores. Cancer Lett. 2014; 353:264–71. 10.1016/j.canlet.2014.07.029 25088578

[119] Giannelli G, Koudelkova P, Dituri F, Mikulits W. Role of epithelial to mesenchymal transition in hepatocellular carcinoma. J Hepatol. 2016; 65:798–808. 10.1016/j.jhep.2016.05.007 27212245

[120] Dooley S, ten Dijke P. TGF-β in progression of liver disease. Cell Tissue Res. 2012; 347:245–56. 10.1007/s00441-011-1246-y 22006249 PMC3250614

[121] Mazzocca A, Fransvea E, Dituri F, Lupo L, Antonaci S, Giannelli G. Down-regulation of connective tissue growth factor by inhibition of transforming growth factor beta blocks the tumor-stroma cross-talk and tumor progression in hepatocellular carcinoma. Hepatology. 2010; 51:523–34. 10.1002/hep.23285 19821534

[122] Giannelli G, Bergamini C, Fransvea E, Sgarra C, Antonaci S. Laminin-5 with transforming growth factor-beta1 induces epithelial to mesenchymal transition in hepatocellular carcinoma. Gastroenterology. 2005; 129:1375–83. 10.1053/j.gastro.2005.09.055 16285938

[123] van Zijl F, Mair M, Csiszar A, Schneller D, Zulehner G, Huber H, Eferl R, Beug H, Dolznig H, Mikulits W. Hepatic tumor-stroma crosstalk guides epithelial to mesenchymal transition at the tumor edge. Oncogene. 2009; 28:4022–33. 10.1038/onc.2009.253 19718050 PMC2900602

[124] Mima K, Hayashi H, Kuroki H, Nakagawa S, Okabe H, Chikamoto A, Watanabe M, Beppu T, Baba H. Epithelial-mesenchymal transition expression profiles as a prognostic factor for disease-free survival in hepatocellular carcinoma: clinical significance of transforming growth factor-β signaling. Oncol Lett. 2013; 5:149–54. 10.3892/ol.2012.954 23255911 PMC3525349

[125] Fransvea E, Angelotti U, Antonaci S, Giannelli G. Blocking transforming growth factor-beta up-regulates E-cadherin and reduces migration and invasion of hepatocellular carcinoma cells. Hepatology. 2008; 47:1557–66. 10.1002/hep.22201 18318443

[126] Fransvea E, Mazzocca A, Antonaci S, Giannelli G. Targeting transforming growth factor (TGF)-betaRI inhibits activation of beta1 integrin and blocks vascular invasion in hepatocellular carcinoma. Hepatology. 2009; 49:839–50. 10.1002/hep.22731 19115199

[127] Mazzocca A, Fransvea E, Lavezzari G, Antonaci S, Giannelli G. Inhibition of transforming growth factor beta receptor I kinase blocks hepatocellular carcinoma growth through neo-angiogenesis regulation. Hepatology. 2009; 50:1140–51. 10.1002/hep.23118 19711426

[128] Fransvea E, Mazzocca A, Santamato A, Azzariti A, Antonaci S, Giannelli G. Kinase activation profile associated with TGF-β-dependent migration of HCC cells: a preclinical study. Cancer Chemother Pharmacol. 2011; 68:79–86. 10.1007/s00280-010-1459-x 20844878

[129] Dituri F, Mazzocca A, Fernando J, Peidrò FJ, Papappicco P, Fabregat I, De Santis F, Paradiso A, Sabbà C, Giannelli G. Differential inhibition of the TGF-β signaling pathway in HCC cells using the small molecule inhibitor LY2157299 and the D10 monoclonal antibody against TGF-β receptor type II. PLoS One. 2013; 8:e67109. 10.1371/journal.pone.0067109 23826206 PMC3694933

[130] Bolanos-Garcia VM. Aurora kinases. Int J Biochem Cell Biol. 2005; 37:1572–77. 10.1016/j.biocel.2005.02.021 15896667

[131] Bayliss R, Burgess SG, McIntyre PJ. Switching Aurora-A kinase on and off at an allosteric site. FEBS J. 2017; 284:2947–54. 10.1111/febs.14069 28342286

[132] Aradottir M, Reynisdottir ST, Stefansson OA, Jonasson JG, Sverrisdottir A, Tryggvadottir L, Eyfjord JE, Bodvarsdottir SK. Aurora A is a prognostic marker for breast cancer arising in BRCA2 mutation carriers. J Pathol Clin Res. 2014; 1:33–40. 10.1002/cjp2.6 27499891 PMC4858119

[133] Koh HM, Jang BG, Hyun CL, Kim YS, Hyun JW, Chang WY, Maeng YH. Aurora kinase A is a prognostic marker in colorectal adenocarcinoma. J Pathol Transl Med. 2017; 51:32–39. 10.4132/jptm.2016.10.17 28013532 PMC5267544

[134] Wang J, Yang S, Zhang H, Song Y, Zhang X, Qian H, Han X, Shi Y. Aurora-A as an independent molecular prognostic marker in gastric cancer. Oncol Rep. 2011; 26:23–32. 10.3892/or.2011.1250 21479365

[135] Liu F, Wang G, Wang X, Che Z, Dong W, Guo X, Wang Z, Chen P, Hou D, Zhang Q, Zhang W, Pan Y, Yang D, et al. Targeting high Aurora kinases expression as an innovative therapy for hepatocellular carcinoma. Oncotarget. 2017; 8:27953–65. 10.18632/oncotarget.15853 28427193 PMC5438621

[136] Tang A, Gao K, Chu L, Zhang R, Yang J, Zheng J. Aurora kinases: novel therapy targets in cancers. Oncotarget. 2017; 8:23937–54. 10.18632/oncotarget.14893 28147341 PMC5410356

[137] Dauch D, Rudalska R, Cossa G, Nault JC, Kang TW, Wuestefeld T, Hohmeyer A, Imbeaud S, Yevsa T, Hoenicke L, Pantsar T, Bozko P, Malek NP, et al. A MYC-aurora kinase A protein complex represents an actionable drug target in p53-altered liver cancer. Nat Med. 2016; 22:744–53. 10.1038/nm.4107 27213815

[138] D’Assoro AB, Haddad T, Galanis E. Aurora-A kinase as a promising therapeutic target in cancer. Front Oncol. 2016; 5:295. 10.3389/fonc.2015.00295 26779440 PMC4701905

[139] González-Loyola A, Fernández-Miranda G, Trakala M, Partida D, Samejima K, Ogawa H, Cañamero M, de Martino A, Martínez-Ramírez Á, de Cárcer G, Pérez de Castro I, Earnshaw WC, Malumbres M. Aurora B overexpression causes aneuploidy and p21Cip1 repression during tumor development. Mol Cell Biol. 2015; 35:3566–78. 10.1128/MCB.01286-14 26240282 PMC4573715

[140] Porcelli L, Guida G, Quatrale AE, Cocco T, Sidella L, Maida I, Iacobazzi RM, Ferretta A, Stolfa DA, Strippoli S, Guida S, Tommasi S, Guida M, Azzariti A. Aurora kinase B inhibition reduces the proliferation of metastatic melanoma cells and enhances the response to chemotherapy. J Transl Med. 2015; 13:26. 10.1186/s12967-015-0385-4 25623468 PMC4314759

[141] Borisa AC, Bhatt HG. A comprehensive review on Aurora kinase: small molecule inhibitors and clinical trial studies. Eur J Med Chem. 2017; 140:1–19. 10.1016/j.ejmech.2017.08.045 28918096

[142] Katayama H, Sen S. Aurora kinase inhibitors as anticancer molecules. Biochim Biophys Acta. 2010; 1799:829–39. 10.1016/j.bbagrm.2010.09.004 20863917 PMC4501772

[143] Girdler F, Gascoigne KE, Eyers PA, Hartmuth S, Crafter C, Foote KM, Keen NJ, Taylor SS. Validating Aurora B as an anti-cancer drug target. J Cell Sci. 2006; 119:3664–75. 10.1242/jcs.03145 16912073

[144] Vakifahmetoglu H, Olsson M, Zhivotovsky B. Death through a tragedy: mitotic catastrophe. Cell Death Differ. 2008; 15:1153–62. 10.1038/cdd.2008.47 18404154

[145] Jeng YM, Peng SY, Lin CY, Hsu HC. Overexpression and amplification of Aurora-A in hepatocellular carcinoma. Clin Cancer Res. 2004; 10:2065–71. 10.1158/1078-0432.CCR-1057-03 15041727

[146] Lin ZZ, Jeng YM, Hu FC, Pan HW, Tsao HW, Lai PL, Lee PH, Cheng AL, Hsu HC. Significance of Aurora B overexpression in hepatocellular carcinoma. Aurora B Overexpression in HCC. BMC Cancer. 2010; 10:461. 10.1186/1471-2407-10-461 20799978 PMC2940801

[147] Zhang K, Wang T, Zhou H, Feng B, Chen Y, Zhi Y, Wang R. A novel aurora-A inhibitor (MLN8237) synergistically enhances the antitumor activity of sorafenib in hepatocellular carcinoma. Mol Ther Nucleic Acids. 2018; 13:176–88. 10.1016/j.omtn.2018.08.014 30292139 PMC6172479

[148] Zhu Q, Yu X, Zhou ZW, Luo M, Zhou C, He ZX, Chen Y, Zhou SF. A quantitative proteomic response of hepatocellular carcinoma Hep3B cells to danusertib, a pan-Aurora kinase inhibitor. J Cancer. 2018; 9:2061–71. 10.7150/jca.20822 29937924 PMC6010685

[149] Lin ZZ, Hsu HC, Hsu CH, Yeh PY, Huang CY, Huang YF, Chen TJ, Kuo SH, Hsu C, Hu FC, Jeng YM, Chung Y, Cheng AL. The Aurora kinase inhibitor VE-465 has anticancer effects in pre-clinical studies of human hepatocellular carcinoma. J Hepatol. 2009; 50:518–27. 10.1016/j.jhep.2008.10.022 19155085

[150] Benten D, Keller G, Quaas A, Schrader J, Gontarewicz A, Balabanov S, Braig M, Wege H, Moll J, Lohse AW, Brummendorf TH. Aurora kinase inhibitor PHA-739358 suppresses growth of hepatocellular carcinoma in vitro and in a xenograft mouse model. Neoplasia. 2009; 11:934–44. 10.1593/neo.09664 19724687 PMC2735802

[151] Aihara A, Tanaka S, Yasen M, Matsumura S, Mitsunori Y, Murakata A, Noguchi N, Kudo A, Nakamura N, Ito K, Arii S. The selective Aurora B kinase inhibitor AZD1152 as a novel treatment for hepatocellular carcinoma. J Hepatol. 2010; 52:63–71. 10.1016/j.jhep.2009.10.013 19913935

[152] Zhang C, Wu X, Zhang M, Zhu L, Zhao R, Xu D, Lin Z, Liang C, Chen T, Chen L, Ren Y, Zhang J, Qin N, Zhang X. Small molecule R1498 as a well-tolerated and orally active kinase inhibitor for hepatocellular carcinoma and gastric cancer treatment via targeting angiogenesis and mitosis pathways. PLoS One. 2013; 8:e65264. 10.1371/journal.pone.0065264 23755206 PMC3673949

[153] Haider C, Grubinger M, Řezníčková E, Weiss TS, Rotheneder H, Miklos W, Berger W, Jorda R, Zatloukal M, Gucky T, Strnad M, Kryštof V, Mikulits W. Novel inhibitors of cyclin-dependent kinases combat hepatocellular carcinoma without inducing chemoresistance. Mol Cancer Ther. 2013; 12:1947–57. 10.1158/1535-7163.MCT-13-0263 23939380

[154] Shen S, Dean DC, Yu Z, Duan Z. Role of cyclin-dependent kinases (CDKs) in hepatocellular carcinoma: therapeutic potential of targeting the CDK signaling pathway. Hepatol Res. 2019; 49:1097–108. 10.1111/hepr.13353 31009153

[155] Roskoski R Jr. Cyclin-dependent protein kinase inhibitors including palbociclib as anticancer drugs. Pharmacol Res. 2016; 107:249–75. 10.1016/j.phrs.2016.03.012 26995305

[156] Bloom J, Cross FR. Multiple levels of cyclin specificity in cell-cycle control. Nat Rev Mol Cell Biol. 2007; 8:149–60. 10.1038/nrm2105 17245415

[157] Roskoski R Jr. Cyclin-dependent protein serine/threonine kinase inhibitors as anticancer drugs. Pharmacol Res. 2019; 139:471–88. 10.1016/j.phrs.2018.11.035 30508677

[158] Bisteau X, Caldez MJ, Kaldis P. The complex relationship between liver cancer and the cell cycle: A story of multiple regulations. Cancers (Basel). 2014; 6:79–111. 10.3390/cancers6010079 24419005 PMC3980619

[159] Matsuda Y, Wakai T, Kubota M, Takamura M, Yamagiwa S, Aoyagi Y, Osawa M, Fujimaki S, Sanpei A, Genda T, Ichida T. Clinical significance of cell cycle inhibitors in hepatocellular carcinoma. Med Mol Morphol. 2013; 46:185–92. 10.1007/s00795-013-0047-7 23640750

[160] Cai J, Li B, Zhu Y, Fang X, Zhu M, Wang M, Liu S, Jiang X, Zheng J, Zhang X, Chen P. Prognostic biomarker identification through integrating the gene signatures of hepatocellular carcinoma properties. EBioMedicine. 2017; 19:18–30. 10.1016/j.ebiom.2017.04.014 28434945 PMC5440601

[161] Kwak MS, Yu SJ, Yoon JH, Lee SH, Lee SM, Lee JH, Kim YJ, Lee HS, Kim CY. Synergistic anti-tumor efficacy of doxorubicin and flavopiridol in an in vivo hepatocellular carcinoma model. J Cancer Res Clin Oncol. 2015; 141:2037–45. 10.1007/s00432-015-1990-6 25989942 PMC11823803

[162] Kohzato N, Dong Y, Sui L, Masaki T, Nagahata S, Nishioka M, Konishi R, Tokuda M. Overexpression of cyclin E and cyclin-dependent kinase 2 is correlated with development of hepatocellular carcinomas. Hepatol Res. 2001; 1:27–39. 10.1016/s1386-6346(00)00150-9 11470626

[163] Qinfeng H, Junhong L, Ailing W. Identification of potential therapeutic targets in hepatocellular carcinoma using an integrated bioinformatics approach. Transl Cancer Res. 2018; 7:849–58. 10.21037/tcr.2018.06.04

[164] Bahnassy AA, Zekri AR, Loutfy SA, Mohamed WS, Moneim AA, Salem SE, Sheta MM, Omar A, Al-Zawahry H. The role of cyclins and cyclin dependent kinases in development and progression of hepatitis C virus-genotype 4-associated hepatitis and hepatocellular carcinoma. Exp Mol Pathol. 2011; 91:643–52. 10.1016/j.yexmp.2011.06.014 21801719

[165] Li KK, Ng IO, Fan ST, Albrecht JH, Yamashita K, Poon RY. Activation of cyclin-dependent kinases CDC2 and CDK2 in hepatocellular carcinoma. Liver. 2002; 22:259–68. 10.1046/j.0106-9543.2002.01629.x 12100577

[166] Cho SJ, Lee SS, Kim YJ, Park BD, Choi JS, Liu L, Ham YM, Moon Kim B, Lee SK. Xylocydine, a novel Cdk inhibitor, is an effective inducer of apoptosis in hepatocellular carcinoma cells in vitro and in vivo. Cancer Lett. 2010; 287:196–206. 10.1016/j.canlet.2009.06.011 19616371

[167] Lu JW, Lin YM, Chang JG, Yeh KT, Chen RM, Tsai JJ, Su WW, Hu RM. Clinical implications of deregulated CDK4 and Cyclin D1 expression in patients with human hepatocellular carcinoma. Med Oncol. 2013; 30:379. 10.1007/s12032-012-0379-5 23292829

[168] Xu XR, Huang J, Xu ZG, Qian BZ, Zhu ZD, Yan Q, Cai T, Zhang X, Xiao HS, Qu J, Liu F, Huang QH, Cheng ZH, et al. Insight into hepatocellular carcinogenesis at transcriptome level by comparing gene expression profiles of hepatocellular carcinoma with those of corresponding noncancerous liver. Proc Natl Acad Sci USA. 2001; 98:15089–94. 10.1073/pnas.241522398 11752456 PMC64988

[169] Zardavas D, Pondé N, Tryfonidis K. CDK4/6 blockade in breast cancer: current experience and future perspectives. Expert Opin Investig Drugs. 2017; 26:1357–72. 10.1080/13543784.2017.1389896 29027483

[170] Bollard J, Miguela V, Ruiz de Galarreta M, Venkatesh A, Bian CB, Roberto MP, Tovar V, Sia D, Molina-Sánchez P, Nguyen CB, Nakagawa S, Llovet JM, Hoshida Y, Lujambio A. Palbociclib (PD-0332991), a selective CDK4/6 inhibitor, restricts tumour growth in preclinical models of hepatocellular carcinoma. Gut. 2017; 66:1286–96. 10.1136/gutjnl-2016-312268 27849562 PMC5512174

[171] Huang CY, Hsieh FS, Wang CY, Chen LJ, Chang SS, Tsai MH, Hung MH, Kuo CW, Shih CT, Chao TI, Chen KF. Palbociclib enhances radiosensitivity of hepatocellular carcinoma and cholangiocarcinoma via inhibiting ataxia telangiectasia-mutated kinase-mediated DNA damage response. Eur J Cancer. 2018; 102:10–22. 10.1016/j.ejca.2018.07.010 30103095

[172] Shah K, Lahiri DK. Cdk5 activity in the brain - multiple paths of regulation. J Cell Sci. 2014; 127:2391–400. 10.1242/jcs.147553 24879856 PMC4038939

[173] Liman J, Deeg S, Voigt A, Voßfeldt H, Dohm CP, Karch A, Weishaupt J, Schulz JB, Bähr M, Kermer P. CDK5 protects from caspase-induced Ataxin-3 cleavage and neurodegeneration. J Neurochem. 2014; 129:1013–23. 10.1111/jnc.12684 24548080

[174] Dixit AB, Banerjee J, Tripathi M, Sarkar C, Chandra PS. Synaptic roles of cyclin-dependent kinase 5 & its implications in epilepsy. Indian J Med Res. 2017; 145:179–88. 10.4103/ijmr.IJMR_1249_14 28639593 PMC5501049

[175] Herzog J, Ehrlich SM, Pfitzer L, Liebl J, Fröhlich T, Arnold GJ, Mikulits W, Haider C, Vollmar AM, Zahler S. Cyclin-dependent kinase 5 stabilizes hypoxia-inducible factor-1α: a novel approach for inhibiting angiogenesis in hepatocellular carcinoma. Oncotarget. 2016; 7:27108–21. 10.18632/oncotarget.8342 27027353 PMC5053636

[176] Ehrlich SM, Liebl J, Ardelt MA, Lehr T, De Toni EN, Mayr D, Brandl L, Kirchner T, Zahler S, Gerbes AL, Vollmar AM. Targeting cyclin dependent kinase 5 in hepatocellular carcinoma—A novel therapeutic approach. J Hepatol. 2015; 63:102–13. 10.1016/j.jhep.2015.01.031 25660209

[177] Zhang R, Lin P, Yang H, He Y, Dang YW, Feng ZB, Chen G. Clinical role and biological function of CDK5 in hepatocellular carcinoma: A study based on immunohistochemistry, RNA-seq and *in vitro* investigation. Oncotarget. 2017; 8:108333–54. 10.18632/oncotarget.22659 29312535 PMC5752448

[178] Ardelt MA, Fröhlich T, Martini E, Müller M, Kanitz V, Atzberger C, Cantonati P, Meßner M, Posselt L, Lehr T, Wojtyniak JG, Ulrich M, Arnold GJ, et al. Inhibition of cyclin-dependent kinase 5: A strategy to improve sorafenib response in hepatocellular carcinoma Therapy. Hepatology. 2019; 69:376–93. 10.1002/hep.30190 30033593 PMC6590289

[179] Peserico A, Simone C. Physical and functional HAT/HDAC interplay regulates protein acetylation balance. J Biomed Biotechnol. 2011; 2011:371832. 10.1155/2011/371832 21151613 PMC2997516

[180] Chen HP, Zhao YT, Zhao TC. Histone deacetylases and mechanisms of regulation of gene expression. Crit Rev Oncog. 2015; 20:35–47. 10.1615/CritRevOncog.2015012997 25746103 PMC4809735

[181] Montezuma D, Henrique RM, Jerónimo C. Altered expression of histone deacetylases in cancer. Crit Rev Oncog. 2015; 20:19–34. 10.1615/CritRevOncog.2014012554 25746102

[182] Tsilimigras DI, Ntanasis-Stathopoulos I, Moris D, Spartalis E, Pawlik TM. Histone deacetylase inhibitors in hepatocellular carcinoma: A therapeutic perspective. Surg Oncol. 2018; 27:611–18. 10.1016/j.suronc.2018.07.015 30449480

[183] Nam SW, Park JY, Ramasamy A, Shevade S, Islam A, Long PM, Park CK, Park SE, Kim SY, Lee SH, Park WS, Yoo NJ, Liu ET, et al. Molecular changes from dysplastic nodule to hepatocellular carcinoma through gene expression profiling. Hepatology. 2005; 42:809–18. 10.1002/hep.20878 16175600

[184] Buurman R, Gürlevik E, Schäffer V, Eilers M, Sandbothe M, Kreipe H, Wilkens L, Schlegelberger B, Kühnel F, Skawran B. Histone deacetylases activate hepatocyte growth factor signaling by repressing microRNA-449 in hepatocellular carcinoma cells. Gastroenterology. 2012; 143:811–820.e15. 10.1053/j.gastro.2012.05.033 22641068

[185] Xie HJ, Noh JH, Kim JK, Jung KH, Eun JW, Bae HJ, Kim MG, Chang YG, Lee JY, Park H, Nam SW. HDAC1 inactivation induces mitotic defect and caspase-independent autophagic cell death in liver cancer. PLoS One. 2012; 7:e34265. 10.1371/journal.pone.0034265 22496786 PMC3319574

[186] Lachenmayer A, Toffanin S, Cabellos L, Alsinet C, Hoshida Y, Villanueva A, Minguez B, Tsai HW, Ward SC, Thung S, Friedman SL, Llovet JM. Combination therapy for hepatocellular carcinoma: additive preclinical efficacy of the HDAC inhibitor panobinostat with sorafenib. J Hepatol. 2012; 56:1343–50. 10.1016/j.jhep.2012.01.009 22322234 PMC3355195

[187] Jiang H, Zhang X, Tao Y, Shan L, Jiang Q, Yu Y, Cai F, Ma L. Prognostic and clinicopathologic significance of SIRT1 expression in hepatocellular carcinoma. Oncotarget. 2016; 8:52357–65. 10.18632/oncotarget.14096 28881735 PMC5581034

[188] Zhou B, Yang Y, Li C. SIRT1 inhibits hepatocellular carcinoma metastasis by promoting M1 macrophage polarization via NF-κB pathway. Onco Targets Ther. 2019; 12:2519–29. 10.2147/OTT.S195234 31040695 PMC6452816

[189] Marquardt JU, Fischer K, Baus K, Kashyap A, Ma S, Krupp M, Linke M, Teufel A, Zechner U, Strand D, Thorgeirsson SS, Galle PR, Strand S. Sirtuin-6-dependent genetic and epigenetic alterations are associated with poor clinical outcome in hepatocellular carcinoma patients. Hepatology. 2013; 58:1054–64. 10.1002/hep.26413 23526469 PMC3759627

[190] Ran LK, Chen Y, Zhang ZZ, Tao NN, Ren JH, Zhou L, Tang H, Chen X, Chen K, Li WY, Huang AL, Chen J. SIRT6 overexpression potentiates apoptosis evasion in hepatocellular carcinoma via BCL2-associated X protein-dependent apoptotic pathway. Clin Cancer Res. 2016; 22:3372–82. 10.1158/1078-0432.CCR-15-1638 26861461

[191] Herold C, Ganslmayer M, Ocker M, Hermann M, Geerts A, Hahn EG, Schuppan D. The histone-deacetylase inhibitor Trichostatin A blocks proliferation and triggers apoptotic programs in hepatoma cells. J Hepatol. 2002; 36:233–40. 10.1016/S0168-8278(01)00257-4 11830335

[192] Chen JC, Chuang HY, Liao YJ, Hsu FT, Chen YC, Wang WH, Hwang JJ. Enhanced cytotoxicity of human hepatocellular carcinoma cells following pretreatment with sorafenib combined with trichostatin A. Oncol Lett. 2019; 17:638–45. 10.3892/ol.2018.9582 30655811 PMC6313190

[193] Shin S, Kim M, Lee SJ, Park KS, Lee CH. Trichostatin A densitizes hepatocellular carcinoma cells to enhanced NK cell-mediated killing by regulating immune-related genes. Cancer Genomics Proteomics. 2017; 14:349–62. 10.21873/cgp.20045 28871002 PMC5611521

[194] Carlisi D, Lauricella M, D’Anneo A, Emanuele S, Angileri L, Di Fazio P, Santulli A, Vento R, Tesoriere G. The histone deacetylase inhibitor suberoylanilide hydroxamic acid sensitises human hepatocellular carcinoma cells to TRAIL-induced apoptosis by TRAIL-DISC activation. Eur J Cancer. 2009; 45:2425–38. 10.1016/j.ejca.2009.06.024 19643600

[195] Liu YL, Yang PM, Shun CT, Wu MS, Weng JR, Chen CC. Autophagy potentiates the anti-cancer effects of the histone deacetylase inhibitors in hepatocellular carcinoma. Autophagy. 2010; 6:1057–65. 10.4161/auto.6.8.13365 20962572

[196] Zhang C, Yang C, Feldman MJ, Wang H, Pang Y, Maggio DM, Zhu D, Nesvick CL, Dmitriev P, Bullova P, Chittiboina P, Brady RO, Pacak K, Zhuang Z. Vorinostat suppresses hypoxia signaling by modulating nuclear translocation of hypoxia inducible factor 1 alpha. Oncotarget. 2017; 8:56110–25. 10.18632/oncotarget.18125 28915577 PMC5593548

[197] Srinivas C, Swathi V, Priyanka C, Anjana Devi T, Subba Reddy BV, Janaki Ramaiah M, Bhadra U, Bhadra MP. Novel SAHA analogues inhibit HDACs, induce apoptosis and modulate the expression of microRNAs in hepatocellular carcinoma. Apoptosis. 2016; 21:1249–64. 10.1007/s10495-016-1278-6 27502208

[198] Liao B, Zhang Y, Sun Q, Jiang P. Vorinostat enhances the anticancer effect of oxaliplatin on hepatocellular carcinoma cells. Cancer Med. 2018; 7:196–207. 10.1002/cam4.1278 29239146 PMC5773972

[199] Di Fazio P, Schneider-Stock R, Neureiter D, Okamoto K, Wissniowski T, Gahr S, Quint K, Meissnitzer M, Alinger B, Montalbano R, Sass G, Hohenstein B, Hahn EG, Ocker M. The pan-deacetylase inhibitor panobinostat inhibits growth of hepatocellular carcinoma models by alternative pathways of apoptosis. Cell Oncol. 2010; 32:285–300. 10.3233/CLO-2010-0511 20208142 PMC4619232

[200] Gahr S, Mayr C, Kiesslich T, Illig R, Neureiter D, Alinger B, Ganslmayer M, Wissniowski T, Fazio PD, Montalbano R, Ficker JH, Ocker M, Quint K. The pan-deacetylase inhibitor panobinostat affects angiogenesis in hepatocellular carcinoma models via modulation of CTGF expression. Int J Oncol. 2015; 47:963–70. 10.3892/ijo.2015.3087 26202945

[201] Soukupova J, Bertran E, Peñuelas-Haro I, Urdiroz-Urricelqui U, Borgman M, Kohlhof H, Fabregat I. Resminostat induces changes in epithelial plasticity of hepatocellular carcinoma cells and sensitizes them to sorafenib-induced apoptosis. Oncotarget. 2017; 8:110367–79. 10.18632/oncotarget.22775 29299154 PMC5746389

[202] Bitzer M, Horger M, Giannini EG, Ganten TM, Wörns MA, Siveke JT, Dollinger MM, Gerken G, Scheulen ME, Wege H, Zagonel V, Cillo U, Trevisani F, et al. Resminostat plus sorafenib as second-line therapy of advanced hepatocellular carcinoma - The SHELTER study. J Hepatol. 2016; 65:280–88. 10.1016/j.jhep.2016.02.043 26952006

[203] Ma BB, Sung F, Tao Q, Poon FF, Lui VW, Yeo W, Chan SL, Chan AT. The preclinical activity of the histone deacetylase inhibitor PXD101 (belinostat) in hepatocellular carcinoma cell lines. Invest New Drugs. 2010; 28:107–14. 10.1007/s10637-009-9219-7 19172229

[204] Spratlin JL, Pitts TM, Kulikowski GN, Morelli MP, Tentler JJ, Serkova NJ, Eckhardt SG. Synergistic activity of histone deacetylase and proteasome inhibition against pancreatic and hepatocellular cancer cell lines. Anticancer Res. 2011; 31:1093–103. 21508352 PMC3866806

[205] Llopiz D, Ruiz M, Villanueva L, Iglesias T, Silva L, Egea J, Lasarte JJ, Pivette P, Trochon-Joseph V, Vasseur B, Dixon G, Sangro B, Sarobe P. Enhanced anti-tumor efficacy of checkpoint inhibitors in combination with the histone deacetylase inhibitor Belinostat in a murine hepatocellular carcinoma model. Cancer Immunol Immunother. 2019; 68:379–93. 10.1007/s00262-018-2283-0 30547218 PMC11028337

[206] Yeo W, Chung HC, Chan SL, Wang LZ, Lim R, Picus J, Boyer M, Mo FK, Koh J, Rha SY, Hui EP, Jeung HC, Roh JK, et al. Epigenetic therapy using belinostat for patients with unresectable hepatocellular carcinoma: a multicenter phase I/II study with biomarker and pharmacokinetic analysis of tumors from patients in the Mayo Phase II Consortium and the Cancer Therapeutics Research Group. J Clin Oncol. 2012; 30:3361–67. 10.1200/JCO.2011.41.2395 22915658 PMC3438233

[207] Iñarrairaegui M, Melero I, Sangro B. Immunotherapy of hepatocellular carcinoma: facts and hopes. Clin Cancer Res. 2018; 24:1518–24. 10.1158/1078-0432.CCR-17-0289 29138342

[208] Ishida Y, Agata Y, Shibahara K, Honjo T. Induced expression of PD-1, a novel member of the immunoglobulin gene superfamily, upon programmed cell death. EMBO J. 1992; 11:3887–95. 10.1002/j.1460-2075.1992.tb05481.x 1396582 PMC556898

[209] Krummel MF, Allison JP. CD28 and CTLA-4 have opposing effects on the response of T cells to stimulation. J Exp Med. 1995; 182:459–65. 10.1084/jem.182.2.459 7543139 PMC2192127

[210] Fife BT, Bluestone JA. Control of peripheral T-cell tolerance and autoimmunity via the CTLA-4 and PD-1 pathways. Immunol Rev. 2008; 224:166–82. 10.1111/j.1600-065X.2008.00662.x 18759926

[211] Fife BT, Pauken KE, Eagar TN, Obu T, Wu J, Tang Q, Azuma M, Krummel MF, Bluestone JA. Interactions between PD-1 and PD-L1 promote tolerance by blocking the TCR-induced stop signal. Nat Immunol. 2009; 10:1185–92. 10.1038/ni.1790 19783989 PMC2778301

[212] Marin-Acevedo JA, Dholaria B, Soyano AE, Knutson KL, Chumsri S, Lou Y. Next generation of immune checkpoint therapy in cancer: new developments and challenges. J Hematol Oncol. 2018; 11:39. 10.1186/s13045-018-0582-8 29544515 PMC5856308

[213] Teft WA, Kirchhof MG, Madrenas J. A molecular perspective of CTLA-4 function. Annu Rev Immunol. 2006; 24:65–97. 10.1146/annurev.immunol.24.021605.090535 16551244

[214] Bristol-Myers Squibb Company FDA Approves YERVOY™. (ipilimumab) for the treatment of patients with newly diagnosed or previously-treated unresectable or metastatic melanoma, the deadliest form of skin cancer. Media Release. https://news.bms.com/press-release/rd-news/fda-approves-yervoy-ipilimumab

[215] Hodi FS, O’Day SJ, McDermott DF, Weber RW, Sosman JA, Haanen JB, Gonzalez R, Robert C, Schadendorf D, Hassel JC, Akerley W, van den Eertwegh AJ, Lutzky J, et al. Improved survival with ipilimumab in patients with metastatic melanoma. N Engl J Med. 2010; 363:711–23. 10.1056/NEJMoa1003466 20525992 PMC3549297

[216] Sangro B, Gomez-Martin C, de la Mata M, Iñarrairaegui M, Garralda E, Barrera P, Riezu-Boj JI, Larrea E, Alfaro C, Sarobe P, Lasarte JJ, Pérez-Gracia JL, Melero I, Prieto J. A clinical trial of CTLA-4 blockade with tremelimumab in patients with hepatocellular carcinoma and chronic hepatitis C. J Hepatol. 2013; 59:81–88. 10.1016/j.jhep.2013.02.022 23466307

[217] Finger LR, Pu J, Wasserman R, Vibhakar R, Louie E, Hardy RR, Burrows PD, Billips LG. The human PD-1 gene: complete cDNA, genomic organization, and developmentally regulated expression in B cell progenitors. Gene. 1997; 197:177–87. 10.1016/S0378-1119(97)00260-6 9332365

[218] Zak KM, Grudnik P, Magiera K, Dömling A, Dubin G, Holak TA. Structural Biology of the Immune Checkpoint Receptor PD-1 and Its Ligands PD-L1/PD-L2. Structure. 2017; 25:1163–74. 10.1016/j.str.2017.06.011 28768162

[219] Gao Q, Wang XY, Qiu SJ, Yamato I, Sho M, Nakajima Y, Zhou J, Li BZ, Shi YH, Xiao YS, Xu Y, Fan J. Overexpression of PD-L1 significantly associates with tumor aggressiveness and postoperative recurrence in human hepatocellular carcinoma. Clin Cancer Res. 2009; 15:971–79. 10.1158/1078-0432.CCR-08-1608 19188168

[220] Calderaro J, Rousseau B, Amaddeo G, Mercey M, Charpy C, Costentin C, Luciani A, Zafrani ES, Laurent A, Azoulay D, Lafdil F, Pawlotsky JM. Programmed death ligand 1 expression in hepatocellular carcinoma: relationship With clinical and pathological features. Hepatology. 2016; 64:2038–46. 10.1002/hep.28710 27359084

[221] Jung HI, Jeong D, Ji S, Ahn TS, Bae SH, Chin S, Chung JC, Kim HC, Lee MS, Baek MJ. Overexpression of PD-L1 and PD-L2 is associated with poor prognosis in patients with hepatocellular carcinoma. Cancer Res Treat. 2017; 49:246–54. 10.4143/crt.2016.066 27456947 PMC5266389

[222] Ma LJ, Feng FL, Dong LQ, Zhang Z, Duan M, Liu LZ, Shi JY, Yang LX, Wang ZC, Zhang S, Ding ZB, Ke AW, Cao Y, et al. Clinical significance of PD-1/PD-Ls gene amplification and overexpression in patients with hepatocellular carcinoma. Theranostics. 2018; 8:5690–702. 10.7150/thno.28742 30555574 PMC6276293

[223] Mocan T, Sparchez Z, Craciun R, Bora CN, Leucuta DC. Programmed cell death protein-1 (PD-1)/programmed death-ligand-1 (PD-L1) axis in hepatocellular carcinoma: prognostic and therapeutic perspectives. Clin Transl Oncol. 2019; 21:702–12. 10.1007/s12094-018-1975-4 30387047

[224] Hui E, Cheung J, Zhu J, Su X, Taylor MJ, Wallweber HA, Sasmal DK, Huang J, Kim JM, Mellman I, Vale RD. T cell costimulatory receptor CD28 is a primary target for PD-1-mediated inhibition. Science. 2017; 355:1428–33. 10.1126/science.aaf1292 28280247 PMC6286077

[225] Barber DL, Wherry EJ, Masopust D, Zhu B, Allison JP, Sharpe AH, Freeman GJ, Ahmed R. Restoring function in exhausted CD8 T cells during chronic viral infection. Nature. 2006; 439:682–87. 10.1038/nature04444 16382236

[226] El-Khoueiry AB, Sangro B, Yau T, Crocenzi TS, Kudo M, Hsu C, Kim TY, Choo SP, Trojan J, Welling TH 3rd, Meyer T, Kang YK, Yeo W, et al. Nivolumab in patients with advanced hepatocellular carcinoma (CheckMate 040): an open-label, non-comparative, phase ½ dose escalation and expansion trial. Lancet. 2017; 389:2492–502. 10.1016/S0140-6736(17)31046-2 28434648 PMC7539326

[227] Zhu AX, Finn RS, Edeline J, Cattan S, Ogasawara S, Palmer D, Verslype C, Zagonel V, Fartoux L, Vogel A, Sarker D, Verset G, Chan SL, et al, and KEYNOTE-224 investigators. Pembrolizumab in patients with advanced hepatocellular carcinoma previously treated with sorafenib (KEYNOTE-224): a non-randomised, open-label phase 2 trial. Lancet Oncol. 2018; 19:940–52. 10.1016/S1470-2045(18)30351-6 29875066

[228] https://investors.merck.com/news/press-release-details/2019/Merck-Provides-Update-on-KEYNOTE-240-a-Phase-3-Study-of-KEYTRUDA-pembrolizumab-in-Previously-Treated-Patients-with-Advanced-Hepatocellular-Carcinoma/default.aspx.

[229] Wainberg ZA, Segal NH, Jaeger D, Lee KH, Marshall J, Antonia SJ, Butler M, Sanborn RE, Nemunaitis JJ, Carlson CA, Finn RS, Jin X, Antal J, et al Safety and clinical activity of durvalumab monotherapy in patients with hepatocellular carcinoma (HCC). J Clin Oncol. 2017; 35:4071 10.1200/JCO.2017.35.15_suppl.4071

[230] Kelley RK, Abou-Alfa GK, Bendell JC, Kim TY, Borad MJ, Yong WP, Morse M, Kang YK, Rebelatto M, Makowsky M, Xiao F, Morris SR, Sangro B. Phase I/II study of durvalumab and tremelimumab in patients with unresectable hepatocellular carcinoma(HCC): phase I safety and efficacy analyses. J Clin Oncolol. 2017; 35(suppl; abstr 4073). 10.1200/JCO.2017.35.15_suppl.4073

[231] https://oncologypro.esmo.org/Meeting-Resources/ESMO-2018-Congress/Updated-safety-and-clinical-activity-results-from-a-Phase-Ib-study-of-atezolizumab-bevacizumab-in-hepatocellular-carcinoma-HCC.

[232] World Health Organization. Proposed working definition of an older person in Africa for the MDS Project. WHO. 2002 http://www.who.int/healthinfo/survey/ageingdefnolder/en/

[233] World Health Organization. What is Healthy Ageing? WHO. http://www.who.int/ageing/healthy-ageing/en/

[234] Carrier P, Debette-Gratien M, Jacques J, Loustaud-Ratti V. Cirrhotic patients and older people. World J Hepatol. 2019; 11:663–77. 10.4254/wjh.v11.i9.678 31598192 PMC6783402

[235] World Health Organization. Healt situation in the European Region. 2018 http://www.euro.who.int/en/data-and-evidence/european-health-report/european-health-report-2018/report-by-chapters/chapter-2-health-situation-in-the-european-region

[236] Pallis AG, Fortpied C, Wedding U, Van Nes MC, Penninckx B, Ring A, Lacombe D, Monfardini S, Scalliet P, Wildiers H. EORTC elderly task force position paper: approach to the older cancer patient. Eur J Cancer. 2010; 46:1502–13. 10.1016/j.ejca.2010.02.022 20227872

[237] Llovet JM, Brú C, Bruix J. Prognosis of hepatocellular carcinoma: the BCLC staging classification. Semin Liver Dis. 1999; 19:329–38. 10.1055/s-2007-1007122 10518312

[238] Galle PR, Forner A, Llovet JM, Mazzaferro V, Piscaglia F, Raoul JL, Schirmacher P, Vilgrain V, and European Association for the Study of the Liver. Electronic address: easloffice@easloffice.eu, and European Association for the Study of the Liver. Management of hepatocellular carcinoma. J Hepatol. 2018; 69:182–236. 10.1016/j.jhep.2018.03.019 29628281

[239] Kozyreva ON, Chi D, Clark JW, Wang H, Theall KP, Ryan DP, Zhu AX. A multicenter retrospective study on clinical characteristics, treatment patterns, and outcome in elderly patients with hepatocellular carcinoma. Oncologist. 2011; 16:310–18. 10.1634/theoncologist.2010-0223 21349948 PMC3228108

[240] Oishi K, Itamoto T, Kohashi T, Matsugu Y, Nakahara H, Kitamoto M. Safety of hepatectomy for elderly patients with hepatocellular carcinoma. World J Gastroenterol. 2014; 20:15028–36. 10.3748/wjg.v20.i41.15028 25386051 PMC4223236

[241] Life expectancy and Healthy life expectancy Data by country.. http://apps.who.int/gho/data/view.main.SDG2016LEXv?lang=en.

[242] Tsujita E, Utsunomiya T, Ohta M, Tagawa T, Matsuyama A, Okazaki J, Yamamoto M, Tsutsui S, Ishida T. Outcome of repeat hepatectomy in patients with hepatocellular carcinoma aged 75 years and older. Surgery. 2010; 147:696–703. 10.1016/j.surg.2009.10.054 20015526

[243] Okamura Y, Sugiura T, Ito T, Yamamoto Y, Ashida R, Uesaka K. The short- and long-term outcomes in elderly patients with hepatocellular carcinoma after curative surgery: A case-controlled study with propensity score matching. Eur Surg Res. 2018; 59:380–90. 10.1159/000494733 30554221

[244] Yamada S, Shimada M, Miyake H, Utsunomiya T, Morine Y, Imura S, Ikemoto T, Mori H, Hanaoka J, Iwahashi S, Saito Y. Outcome of hepatectomy in super-elderly patients with hepatocellular carcinoma. Hepatol Res. 2012; 42:454–58. 10.1111/j.1872-034X.2011.00952.x 22295877

[245] Nishikawa H, Arimoto A, Wakasa T, Kita R, Kimura T, Osaki Y. Surgical resection for hepatocellular carcinoma: clinical outcomes and safety in elderly patients. Eur J Gastroenterol Hepatol. 2013; 25:912–19. 10.1097/MEG.0b013e32835fa668 23470356

[246] Ueno M, Hayami S, Tani M, Kawai M, Hirono S, Yamaue H. Recent trends in hepatectomy for elderly patients with hepatocellular carcinoma. Surg Today. 2014; 44:1651–59. 10.1007/s00595-013-0739-6 24091862

[247] Kishida N, Hibi T, Itano O, Okabayashi K, Shinoda M, Kitago M, Abe Y, Yagi H, Kitagawa Y. Validation of hepatectomy for elderly patients with hepatocellular carcinoma. Ann Surg Oncol. 2015; 22:3094–101. 10.1245/s10434-014-4350-x 25582743

[248] Santambrogio R, Barabino M, Scifo G, Costa M, Giovenzana M, Opocher E. Effect of age (over 75 years) on postoperative complications and survival in patients undergoing hepatic resection for hepatocellular carcinoma. J Gastrointest Surg. 2017; 21:657–65. 10.1007/s11605-016-3354-1 28083840

[249] Wu FH, Shen CH, Luo SC, Hwang JI, Chao WS, Yeh HZ, Jan YG, Yen Y, Cheng SB, Wu CC, Lin YL, P’eng FK. Liver resection for hepatocellular carcinoma in oldest old patients. World J Surg Oncol. 2019; 17:1. 10.1186/s12957-018-1541-0 30606220 PMC6317186

[250] Horiuchi T, Haruki K, Shiba H, Sakamoto T, Saito N, Shirai Y, Iwase R, Fujiwara Y, Yanaga K. Assessment of Outcome of Hepatic Resection for Extremely Elderly Patients With a Hepatic Malignancy. Anticancer Res. 2019; 39:6325–32. 10.21873/anticanres.13843 31704863

[251] Kaibori M, Yoshii K, Hasegawa K, Ogawa A, Kubo S, Tateishi R, Izumi N, Kadoya M, Kudo M, Kumada T, Sakamoto M, Nakashima O, Matsuyama Y, et al, and Liver Cancer Study Group of Japan. Treatment Optimization for Hepatocellular Carcinoma in Elderly Patients in a Japanese Nationwide Cohort. Ann Surg. 2019; 270:121–30. 10.1097/SLA.0000000000002751 29608544

[252] Lee CW, Chan KM, Tsai HI, Hsieh YC, Lin CY, Kuo YC, Hsu HY, Yu MC. Hepatic resection for hepatocellular carcinoma in the octogenarian: is it justified? Aging (Albany NY). 2019; 11:1537–50. 10.18632/aging.101854 30867335 PMC6428089

[253] Sandonato L, Soresi M, Cipolla C, Bartolotta TV, Giannitrapani L, Antonucci M, Galia M, Latteri MA. Minor hepatic resection for hepatocellular carcinoma in cirrhotic patients: kelly clamp crushing resection versus heat coagulative necrosis with bipolar radiofrequency device. Am Surg. 2011; 77:1490–95. 22196663

[254] Takahashi H, Mizuta T, Kawazoe S, Eguchi Y, Kawaguchi Y, Otuka T, Oeda S, Ario K, Iwane S, Akiyama T, Ozaki I, Fujimoto K. Efficacy and safety of radiofrequency ablation for elderly hepatocellular carcinoma patients. Hepatol Res. 2010; 40:997–1005. 10.1111/j.1872-034X.2010.00713.x 20887335

[255] Hiraoka A, Michitaka K, Horiike N, Hidaka S, Uehara T, Ichikawa S, Hasebe A, Miyamoto Y, Ninomiya T, Sogabe I, Ishimaru Y, Kawasaki H, Koizumi Y, et al. Radiofrequency ablation therapy for hepatocellular carcinoma in elderly patients. J Gastroenterol Hepatol. 2010; 25:403–07. 10.1111/j.1440-1746.2009.06037.x 19929922

[256] Nishikawa H, Osaki Y, Iguchi E, Takeda H, Ohara Y, Sakamoto A, Hatamaru K, Henmi S, Saito S, Nasu A, Kita R, Kimura T. Percutaneous radiofrequency ablation for hepatocellular carcinoma: clinical outcome and safety in elderly patients. J Gastrointestin Liver Dis. 2012; 21:397–405. 23256123

[257] Schullian P, Putzer D, Silva MA, Laimer G, Kolbitsch C, Bale R. Stereotactic Radiofrequency Ablation of Liver Tumors in Octogenarians. Front Oncol. 2019; 9:929. 10.3389/fonc.2019.00929 31608232 PMC6761359

[258] Teratani T, Ishikawa T, Shiratori Y, Shiina S, Yoshida H, Imamura M, Obi S, Sato S, Hamamura K, Omata M. Hepatocellular carcinoma in elderly patients: beneficial therapeutic efficacy using percutaneous ethanol injection therapy. Cancer. 2002; 95:816–23. 10.1002/cncr.10735 12209726

[259] Mondazzi L, Bottelli R, Brambilla G, Rampoldi A, Rezakovic I, Zavaglia C, Alberti A, Idèo G. Transarterial oily chemoembolization for the treatment of hepatocellular carcinoma: a multivariate analysis of prognostic factors. Hepatology. 1994; 19:1115–23. 10.1002/hep.1840190508 7513677

[260] Cohen MJ, Bloom AI, Barak O, Klimov A, Nesher T, Shouval D, Levi I, Shibolet O. Trans-arterial chemo-embolization is safe and effective for very elderly patients with hepatocellular carcinoma. World J Gastroenterol. 2013; 19:2521–28. 10.3748/wjg.v19.i16.2521 23674854 PMC3646143

[261] Nishikawa H, Kita R, Kimura T, Ohara Y, Takeda H, Sakamoto A, Saito S, Nishijima N, Nasu A, Komekado H, Osaki Y. Transcatheter arterial chemoembolization for intermediate-stage hepatocellular carcinoma: clinical outcome and safety in elderly patients. J Cancer. 2014; 5:590–97. 10.7150/jca.9413 25057310 PMC4107235

[262] Cheng HM, Tanaka T, Nishiofuku H, Chanoki Y, Horiuchi K, Masada T, Tatsumoto S, Matsumoto T, Marugami N, Kichikawa K. Safety and Prognosis of Transarterial Chemoembolization for Octogenarians with Hepatocellular Carcinoma. Cardiovasc Intervent Radiol. 2019; 42:1413–19. 10.1007/s00270-019-02290-x 31338551

[263] Llovet JM, Ricci S, Mazzaferro V, Hilgard P, Gane E, Blanc JF, de Oliveira AC, Santoro A, Raoul JL, Forner A, Schwartz M, Porta C, Zeuzem S, et al, and SHARP Investigators Study Group. Sorafenib in advanced hepatocellular carcinoma. N Engl J Med. 2008; 359:378–90. 10.1056/NEJMoa0708857 18650514

[264] Cheng AL, Kang YK, Chen Z, Tsao CJ, Qin S, Kim JS, Luo R, Feng J, Ye S, Yang TS, Xu J, Sun Y, Liang H, et al. Efficacy and safety of sorafenib in patients in the Asia-Pacific region with advanced hepatocellular carcinoma: a phase III randomised, double-blind, placebo-controlled trial. Lancet Oncol. 2009; 10:25–34. 10.1016/S1470-2045(08)70285-7 19095497

[265] Lencioni R, Kudo M, Ye SL, Bronowicki JP, Chen XP, Dagher L, Furuse J, Geschwind JF, de Guevara LL, Papandreou C, Takayama T, Yoon SK, Nakajima K, et al. GIDEON (Global Investigation of therapeutic DEcisions in hepatocellular carcinoma and Of its treatment with sorafeNib): second interim analysis. Int J Clin Pract. 2014; 68:609–17. 10.1111/ijcp.12352 24283303 PMC4265239

[266] Zolfino T, Lorusso V, D’Angelo S, Sansonno D, Giannitrapani L, Benedetti A, Montesarchio V, Attili AF, Buonadonna A, Barni S, Gasbarrini A, Pirisi M, Cillo U, et al Hepatocellular Carcinoma in Elderly Patients: final results of the Italian Cohort Of GIDEON (Global Investigation of therapeutic Decision and Of its treatment with sorafeNib) Study. Ann Oncol. 2015 (Suppl 6); 26:vi90–105. 10.1093/annonc/mdv344.11

[267] Benson AB, D’Angelica MI, Abbott DE, Abrams TA, Alberts SR, Anaya DA, Anders R, Are C, Brown D, Chang DT, Cloyd J, Covey AM, Hawkins W, et al. Guidelines insights: hepatobiliary cancers, version 2.2019. J Natl Compr Canc Netw. 2019; 17:302–10. 10.6004/jnccn.2019.0019 30959462

[268] Tada T, Kumada T, Hiraoka A, Michitaka K, Atsukawa M, Hirooka M, Tsuji K, Ishikawa T, Takaguchi K, Kariyama K, Itobayashi E, Tajiri K, Shimada N, et al. Safety and efficacy of lenvatinib in elderly patients with unresectable hepatocellular carcinoma: A multicenter analysis with propensity score matching. Hepatol Res. 2019. [Epub ahead of print]. 10.1111/hepr.13427 31660700

